# Performance of international phenotypic criteria for prenatal exome sequencing: systematic review and comparative diagnostic accuracy study using historical individual participant data

**DOI:** 10.1002/uog.29290

**Published:** 2025-07-08

**Authors:** K. Reilly, D. L. Rolnik, S. Allen, A. Sotiriadis, S. Ong, S. Sonner, G. V. Blayney, M. Fernando, T. Van Mieghem, A. Borrell, S. Langlois, F. Mone, Collaborators, A. Khalil, U. Agarwal, S. Nanda, K. W. Choy, Y. Yaron, R. J. Martinez‐Portilla, F. R. Grati, E. Westenius, E. Iwarsson, K. O. Kagan, R. Pooh

**Affiliations:** ^1^ Centre for Public Health Queen's University Belfast Belfast UK; ^2^ Department of Obstetrics and Gynaecology Monash University Melbourne VIC Australia; ^3^ West Midlands Regional Genetics Laboratory Birmingham Women's and Children's NHS Foundation Trust Birmingham UK; ^4^ Second Department of Obstetrics and Gynecology, Faculty of Medicine Aristotle University of Thessaloniki Thessaloniki Greece; ^5^ Centre for Fetal Medicine, Royal Jubilee Maternity Service, Belfast Health and Social Care Trust Belfast UK; ^6^ King's College London Medical School London UK; ^7^ Division of Maternal and Fetal Medicine, Department of Obstetrics and Gynaecology Mount Sinai Hospital, University of Toronto Toronto ON Canada; ^8^ Barcelona Centre for Maternal–Fetal and Neonatal Medicine (BCNatal), Hospital Clínic Barcelona Universitat de Barcelona Barcelona Spain; ^9^ Department of Medical Genetics University of British Columbia Vancouver BC Canada

**Keywords:** criteria, exome sequencing, fetal anomaly, next‐generation sequencing, phenotype, prenatal

## Abstract

**Objectives:**

To evaluate: (i) the performance of the National Health Service (NHS) phenotypic eligibility criteria for prenatal exome sequencing (pES); (ii) the diagnostic yield of individual NHS criteria; (iii) the diagnostic yield when one or multiple NHS criteria were met; and (iv) the performance of the NHS criteria compared with that of phenotypic eligibility criteria used in other countries/regions.

**Methods:**

An online survey was circulated to healthcare professionals in 120 countries to gather information on whether pES is offered in their country and how case selection is performed. Five predefined sets of phenotypic eligibility criteria from England, Greece, Canada (British Columbia and Ontario) and Spain were tested on a virtual historical cohort of 1054 ‘unselected’ structurally abnormal fetuses undergoing pES, derived from a published systematic review. The performance of the current and previous gene panels used in the NHS pES service was assessed in the unselected cohort. The sensitivity and specificity with 95% CI for each set of criteria in relation to diagnostic yield for pathogenic and likely pathogenic variants were calculated, along with the area under the summary receiver‐operating‐characteristics curve (AUC).

**Results:**

The electronic survey received 261 responses from 63/120 countries. Where deducible, 81.8% (45/55) of the countries surveyed offered pES. Where stated, most (90.3% (28/31)) cases were selected for pES on a case‐by‐case basis, according to fetal phenotype and the likelihood of an association with a monogenic condition, rather than on the basis of predetermined phenotypic criteria. The total diagnostic yield of the NHS criteria when applied to all relevant cases for pES was 27.8% (69/248), with pooled sensitivity, pooled specificity and AUC of 49.8% (95% CI, 31.7–67.9%), 80.7% (95% CI, 59.6–92.2%) and 0.66 (95% CI, 0.53–0.74), respectively. The diagnostic yield was highest for isolated short long bones (58.3% (7/12)). The likelihood of a monogenic diagnosis did not increase significantly as the number of NHS criteria met increased. There was a significant increase in the diagnostic yield of the current (2024) *vs* original (2020) gene panel adopted by the NHS (129/135 (95.6%) *vs* 118/135 (87.4%); *P* = 0.017). The four other sets of phenotypic criteria used in other countries/regions performed moderately well, with the best performance seen for the British Columbia criteria, which had a pooled sensitivity of 70.5% (95% CI, 43.7–88.1%), pooled specificity of 68.9% (95% CI, 37.5–89.1%) and AUC of 0.73 (95% CI, 0.58–0.79).

**Conclusions:**

In the majority of countries for which there was a survey response, pES was offered on a case‐by‐case basis, according to fetal phenotype and the likelihood of an underlying monogenic condition. Existing phenotypic eligibility criteria for pES performed modestly. © 2025 The Author(s). *Ultrasound in Obstetrics & Gynecology* published by John Wiley & Sons Ltd on behalf of International Society of Ultrasound in Obstetrics and Gynecology.

## INTRODUCTION

The fetal phenotype represents the structural or physical characteristics of a fetus resulting from the interaction between genotype and environment. In the context of dysmorphology, deep phenotyping refers to the mapping of such structural and physical characteristics based on targeted ultrasound imaging, with the addition of magnetic resonance imaging, three‐dimensional ultrasound imaging, postmortem examination and/or investigation of the phenotypes of affected family members^1^. Accurate phenotyping plays a vital role in all steps of the genomic prenatal diagnostic pathway, including classification, selection and bioinformatic analysis^2^. Although prenatal exome sequencing (pES) is a rapidly evolving tool for genomic investigation with increasing availability worldwide, it remains an expensive resource in publicly funded healthcare systems. Therefore, in such settings, only fetuses exhibiting a pattern of congenital anomalies with a likely monogenic etiology are selected for testing with pES, with a view to optimizing cost‐effectiveness and minimizing variants of uncertain significance (VUS) or incidental findings.

The National Health Service (NHS) in England uses internationally recognized criteria to determine which fetuses are eligible for testing with pES. These selection criteria are updated regularly on the basis of systematic reviews and expert consensus^3^, ^4^, ^5^. This nationally commissioned service uses trio pES with analysis of only a panel of genes, referred to as the R21 fetal anomaly panel, available on PanelApp^6^. In most cases, the analysis runs in parallel with chromosome microarray analysis (CMA), and has an average turnaround time of 21 days. The gene panel is updated regularly based on newly published genotype–phenotype associations.

Other countries and regions use different phenotypic criteria as an indication for pES, and some have no specific criteria^7^, ^8^. Moreover, alternative gene panels or a gene‐agnostic analytical approach may be used. Robust evidence‐based phenotypic eligibility criteria have been shown to be effective for the pES service in the UK, but they have not been compared with other international criteria, nor have other approaches to the prenatal diagnosis of monogenic disorders been assessed^9^, ^10^.

This study aimed to evaluate: (i) the performance of NHS criteria for pES, with regard to the phenotypic eligibility criteria and the gene panel applied for analysis; (ii) the diagnostic yield of individual NHS criteria for pathogenic and likely pathogenic variants; (iii) the diagnostic yield when one, two, or three or more NHS criteria were met; and (iv) the performance of the NHS criteria compared with that of the phenotypic eligibility criteria used in other countries/regions.

## METHODS

### International service evaluation

An international service evaluation was conducted between 4 December 2024 and 1 January 2025. An electronic survey was designed requesting information on country of clinical practice, if pES (or equivalent) is offered and, if so, how cases are selected for pES. A link to the electronic survey was distributed to affiliated healthcare professionals in 120 countries via the GEFOG Health Foundation website (https://gefoghealthfoundation.org/). Consent was obtained at the outset and responses (including IP address) remained anonymous. A link to the survey was circulated twice and remained open for the duration of the service evaluation.

### Virtual historical cohort

Where phenotypic criteria for a country/region were identified, their performance was tested on an ‘unselected’ cohort of fetuses with structural anomaly undergoing pES (i.e. cases had not undergone previous review by a clinical genetics team to ascertain the risk of a monogenic etiology). This dataset was derived from the systematic review of Mellis *et al*.^3^, which, to our knowledge, is the largest published systematic review of pES to date and included 23 original studies in which pES was performed for a fetal structural anomaly following a normal karyotype and/or CMA. Further eligibility criteria were applied in order to include only data from studies in which: (i) the prenatal phenotype was described and pES testing was performed based on this; (ii) both positive and negative pES phenotypic data were included; and (iii) a whole exome or clinical exome of > 1000 genes was applied. Individual participant data (IPD) for the fetuses in this virtual cohort were obtained subsequently from the primary publication and/or supplementary data, including phenotype, gestational age at testing, nature of sequencing (i.e. clinical‐exome, whole‐exome or whole‐genome analysis and single, duo or trio testing), genotype and variant classification. This study was registered prospectively with the International Prospective Register of Systematic Reviews (PROSPERO) (ref. CRD42024602529).

### NHS R21 gene panel

The latest version of the NHS R21 gene panel at the time of writing was v5.0, which includes 1417 ‘green’ genes most relevant to fetal anomalies^10^. A green rating on PanelApp requires evidence of pathogenicity and causation from three or more unrelated families, or from two or three unrelated families where there is strong additional functional data. Variants identified in the genes on the panel are classified according to standardized criteria and, in the majority of cases, only Class‐4 (likely pathogenic) or Class‐5 (pathogenic) variants are reported in a prenatal setting^11^, ^12^. The iterative NHS R21 green gene panels from August 2020 (*n* = 974 genes; v1.92), November 2022 (*n* = 1224 genes; v2.0), March 2023 (*n* = 1239 genes; v3.0), May 2024 (*n* = 1279 genes; v4.0) and October 2024 (*n* = 1417 genes; v5.0) were assessed against the causative pathogenic and likely pathogenic variants identified within the IPD dataset. The performance of sequential NHS panels was compared using the chi‐square test.

### Statistical analysis

The virtual historical cohort derived from the systematic review of Mellis *et al*.^3^ was used to assess the diagnostic yield of the NHS R21 v5.0 phenotypic eligibility criteria, as well as the respective yield for each individual NHS R21 criterion. Subsequently, relevant phenotypic eligibility criteria from other counties/regions were applied to the cohort phenotypes to determine where: (i) phenotyping criteria were met and a causative variant was identified (true positive); (ii) phenotyping criteria were met and no causative variant was identified (false positive); (iii) phenotyping criteria were not met and no causative variant was identified (true negative); and (iv) phenotyping criteria were not met and a causative variant was identified (false negative). Criteria were applied independently by two fetal medicine experts (K.R., S.O.) and discrepancies were checked by a senior researcher (F.M.).

The sensitivity and specificity with 95% CI of each set of criteria in detecting pathogenic and likely pathogenic variants were summarized using forest plots depicting univariate analyses. Heterogeneity was assessed using the *I* ^2^ statistic obtained from random‐effects models using the DerSimonian–Laird procedure and inverse‐variance weighting. A continuity correction of 0.5 was applied when zero cells were present in two‐by‐two tables. For each set of criteria, sensitivity and specificity were pooled using bivariate models as described by Reitsma *et al*.^13^ and following a two‐stage IPD meta‐analysis framework. Summary receiver‐operating‐characteristics (sROC) curves were used to represent graphically the individual studies and the pooled estimates with a 95% confidence region using each set of phenotypic criteria. The areas under the sROC curves (AUC) for each set of phenotypic criteria were compared using bootstrapping, and the pooled sensitivities and specificities were compared using *Z*‐tests. Studies including only fetuses with a unisystem malformation that served as an eligibility criterion for pES in a particular country/region (e.g. any congenital heart defect (CHD)) were omitted from the final meta‐analysis of diagnostic accuracy for that country/region. This decision was taken because including such studies would significantly overestimate sensitivity and underestimate specificity, introducing bias to the pooled estimates.

Statistical analysis was performed using R, version 4.2.1 (R Foundation for Statistical Computing, Vienna, Austria), and the packages ‘meta’ and ‘mada’ were used for univariate analysis and fitting of the bivariate models, respectively^14^, ^15^.

## RESULTS

### International survey

The electronic survey received 261 responses from 63/120 (52.5%) countries (Table S1), with a median of 2 (range, 1–36) respondents per country. With respect to whether pES was offered, there was a discrepancy in response in 28.6% (18/63) of countries, of which eight were excluded from analysis. Where deducible from the majority, pES was offered in 81.8% (45/55) of countries. With respect to how cases were selected for pES, there was a discrepancy in response in 31.1% (14/45) of countries, precluding their inclusion in the analysis. Among the 31 countries for which reported pES criteria were consistent between survey respondents, two (6.5%) offered pES to any fetus with or without an anomaly, one (3.2%) had an established set of phenotypic eligibility criteria and 28 (90.3%) selected fetuses on a case‐by‐case basis, according to the fetal phenotype and the likelihood of an association with a monogenic condition.

### Cohort selection

Of the 23 studies included in the systematic review of Mellis *et al*.^3^, 10 (43.5%) met the inclusion criteria for our analysis^16^, ^17^, ^18^, ^19^, ^20^, ^21^, ^22^, ^23^, ^24^, ^25^. After removing those cases for which the phenotype was not definitive, 1054/1096 (96.2%) individual cases were included (Figure S1). The justification for study selection and the characteristics of the cohort in each included publication are presented in Tables S2 and S3, respectively. In total, 135 causative diagnostic pathogenic or likely pathogenic variants were identified, giving an overall diagnostic yield of 12.8% (95% CI, 11.0–15.1%). Multisystem anomalies were present in 13.1% (138/1054) of the cohort.

### Performance of NHS R21 phenotypic criteria

The NHS R21 criteria were applicable to 248 (23.5%) cases (Tables 1 and 2). The total diagnostic yield of the NHS R21 criteria when applied to all relevant cases for pES, over and above CMA/karyotyping, was 27.8% (69/248), with a pooled sensitivity of 49.8% (95% CI, 31.7–67.9%), pooled specificity of 80.7% (95% CI, 59.6–92.2%) and AUC of 0.66 (95% CI, 0.53–0.74). The diagnostic yields for the individual criteria within the NHS R21 set were 21.7% (30/138) for multisystem anomalies, 33.3% (5/15) for multiple contractures, 25.9% (7/27) for suspected skeletal dysplasia, 42.1% (8/19) for large bilateral echogenic kidneys with normal bladder, 31.3% (20/64) for major central nervous system (CNS) anomaly, 41.7% (5/12) for isolated non‐immune hydrops fetalis (NIHF) and 58.3% (7/12) for isolated short long bones (SLB). There were too few cases to deduce the yield in the other phenotypic subgroups. Despite a trend of increasing yield, there was no significant difference in the likelihood of a single‐gene diagnosis following normal CMA/karyotype when one, two, or three or more criteria were met (26.7%, 33.3% and 37.5%, respectively (*P* = 0.63)).

**Table 1:** Summary of fetal phenotypic eligibility criteria for prenatal exome sequencing in five countries/regions

Body/region	Fetal phenotypic criteria
NHS, UK^10^	Multisystem anomalies Suspected skeletal dysplasia (excluding confirmed *FGFR3/2* variant) Large bilateral echogenic kidneys with normal bladder Major CNS anomaly (excluding NTD) Multiple contractures (excluding isolated bilateral talipes) NT > 6.5 mm plus another anomaly Isolated NIHF detected at or after routine 18–20‐week scan in second or third trimester, defined as fluid/edema in at least two compartments Anomaly of corpus callosum SGA (all biometry < 3^rd^ centile) and no evidence of placental insufficiency Isolated SLB (all < 3^rd^ centile) and no evidence of placental insufficiency
Spanish Association of Prenatal Diagnosis, Spain^7^	Bilateral hyperechogenic, dysplastic or polycystic kidneys Skeletal dysplasia Recurrent anomaly Fetal akinesia deformation sequence Craniosynostosis Multiple anomalies involving various systems Non‐isolated increased NT NIHF Single CNS anomaly Severe early‐onset FGR Isolated congenital heart defect
Ontario Ministry of Health, Canada*	Multiple anomalies: (1) at least two are major and involve at least two different systems; (2) differential diagnosis includes two or more well‐defined conditions requiring evaluation with multiple targeted gene panels; or (3) fetus presents with one major anomaly and FGR not explained by placental or non‐genetic factors Single organ‐system anomaly: (1) two anomalies in brain; (2) two different skeletal findings; or (3) NIHF (two or more fetal compartments, including NT > 3.5 mm) Major anomaly cannot include gastroschisis, isolated NTD or soft markers Anomalies should not present as a highly recognizable pattern specific to a known genetic condition for which an optimized gene panel exists
British Columbia, Canada†	Multiple anomalies (including abnormal growth or amniotic fluid level in the presence of a structural anomaly) Structural brain abnormality (e.g. ACC), microcephaly (HC < 3 SD) or severe ventriculomegaly NIHF Radial ray abnormality Ectrodactyly Skeletal findings suggestive of skeletal dysplasia Bilateral clubfoot Bilateral echogenic kidneys Omphalocele Heterotaxy Congenital heart defect (excluding TGA, TOF and muscular VSD) Ocular findings suggestive of monogenic disorder, such as cataract Abnormal genitalia with discrepancy with NIPT
Hellenic Society of Ultrasound in Obstetrics and Gynecology, Greece^8^	Multiple anomalies Findings suspicious of skeletal dysplasia (unrelated to FGR) Bilateral echogenic kidneys, especially when enlarged with normal bladder Major CNS anomaly (excluding NTD) Major congenital heart disease Multiple contractures/arthrogryposis (excluding isolated bilateral clubfoot) Apparently unexplained fetal overgrowth (all biometry > 97^th^ centile) Isolated NIHF NT > 4 mm at 11–14 weeks Persistent increased nuchal fold (> 6 mm) in second trimester plus structural anomaly in two or more systems Anomaly of corpus callosum Isolated significant FGR of non‐placental origin (biometry < 1^st^ centile, absence of obvious anatomical anomaly, normal Doppler waveforms) Recurrence of an anomaly

* See Appendix S1.

† Criteria obtained by personal communication.

ACC, absent corpus callosum; CNS, central nervous system; FGR, fetal growth restriction; HC, head circumference; NHS, National Health Service; NIHF, non‐immune hydrops fetalis; NIPT, non‐invasive prenatal testing; NT, nuchal translucency; NTD, neural tube defect; SGA, small‐for‐gestational age; SLB, short long bones; TGA, transposition of the great arteries; TOF, tetralogy of Fallot; VSD, ventricular septal defect.

**Table 2:** Performance of international fetal phenotypic eligibility criteria for prenatal exome sequencing in virtual historical cohort (*n* = 1054)

Body/region	Cases sequenced*	Diagnostic yield*	Pooled sensitivity (%)	Pooled specificity (%)	LR+	LR−	Diagnostic odds ratio	AUC	*P* †
NHS, UK^10^	248/1054 (23.5)	69/248 (27.8)	49.8 (31.7–67.9)	80.7 (59.6–92.2)	2.70 (1.52–4.77)	0.63 (0.47–0.78)	4.13 (2.17–7.86)	0.66 (0.53–0.74)	0.723
Spanish Association of Prenatal Diagnosis, Spain^7^	811/1054 (76.9)	122/811 (15.0)	70.8 (52.7–84.1)	63.5 (25.9–89.6)	2.31 (1.03–5.92)	0.51 (0.29–0.92)	4.21 (1.12–15.80)	0.71 (0.57–0.78)	0.345
Ontario Ministry of Health, Canada‡	199/1054 (18.9)	60/199 (30.2)	41.5 (22.1–64.0)	88.1 (72.0–95.5)	3.67 (1.79–7.02)	0.67 (0.45–0.85)	5.24 (2.33–11.80)	0.70 (0.49–0.80)	0.490
British Columbia, Canada§	421/1054 (40.0)	113/421 (26.8)	70.5 (43.7–88.1)	68.9 (37.5–89.1)	2.45 (1.30–4.93)	0.45 (0.23–0.71)	5.30 (2.13–13.20)	0.73 (0.58–0.79)	0.244
Hellenic Society of Ultrasound in Obstetrics and Gynecology, Greece^8^	464/1054 (44.0)	115/464 (24.8)	74.8 (51.0–89.5)	44.4 (25.0–65.7)	1.36 (1.07–1.79)	0.58 (0.33–0.89)	2.38 (1.23–4.61)	0.63 (0.49–0.75)	Reference

Data in parentheses are 95% CI, unless stated otherwise.

* Data are given as *n/N* (%).

† *P*‐values compare areas under summary receiver‐operating‐characteristics curves (AUC) between each set of phenotypic eligibility criteria, with criteria showing lowest AUC selected as reference; AUCs were estimated using 5000 bootstrap samples.

‡ See Appendix S1.

§ Criteria obtained by personal communication.

LR+, positive likelihood ratio; LR−, negative likelihood ratio; NHS, National Health Service.

Regarding the performance of the fetal anomaly gene panel, the first version (v1.92) of the panel would have detected 118/135 (87.4%) variants/diagnoses, whereas the current version (v5.0) of the panel would have detected 129/135 (95.6%) (Table S4), representing a significant increase in diagnostic yield (*P* = 0.017). Eight of the diagnoses reported were in genes that were added to v2.0 of the panel, two in genes added to v3.0, and one in a gene added to v5.0, all as a result of new reports of genotype–phenotype associations. Causative variants detected in genes not present within the current version of the gene panel included two cases of *CACNA1D* and one case each of *ATP7B*, *PPM1D*, *ECE1* and *DNAH7*, and these were consequently reviewed (Table S5). For most of these genes, no clear gene–disease association was reported (*ECE1* and *DNAH7*), or there was no evidence of an association with fetal structural anomaly (*ATP7B* and *PPM1D*). Therefore, the genes did not fulfil the criteria to be included on the fetal anomaly panel.

### Performance of international phenotypic criteria

Due to inconsistencies in reporting within the survey, we compared the performance of previously established phenotypic criteria from four countries/regions to that of the NHS R21 criteria^7^, ^8^, ^10^ (Table 1). Although these countries/regions used defined eligibility criteria for pES, each country/region stipulated that cases could also be reviewed on an individual basis by a multidisciplinary team to determine suitability outside the inclusion criteria. One region (Ontario, Canada) has updated its phenotypic criteria since our analysis was performed; our analysis refers to those provided in Appendix S1.

Of the studies contributing to the virtual historical cohort, the studies of Sun *et al*.^22^, Qiao *et al*.^25^ and Li *et al*.^18^ selected only fetuses with CHD, while that of Yang *et al*.^19^ selected only fetuses with isolated increased nuchal translucency. Hence, these studies were excluded from the analysis of diagnostic accuracy of the eligibility criteria used in Greece (which recommends pES for major CHD and isolated increased nuchal translucency > 4 mm), British Columbia (which recommends pES for CHD except for transposition of the great arteries, tetralogy of Fallot and muscular ventricular septal defect) and Spain (which recommends pES for all isolated CHD).

**Figure 1:**
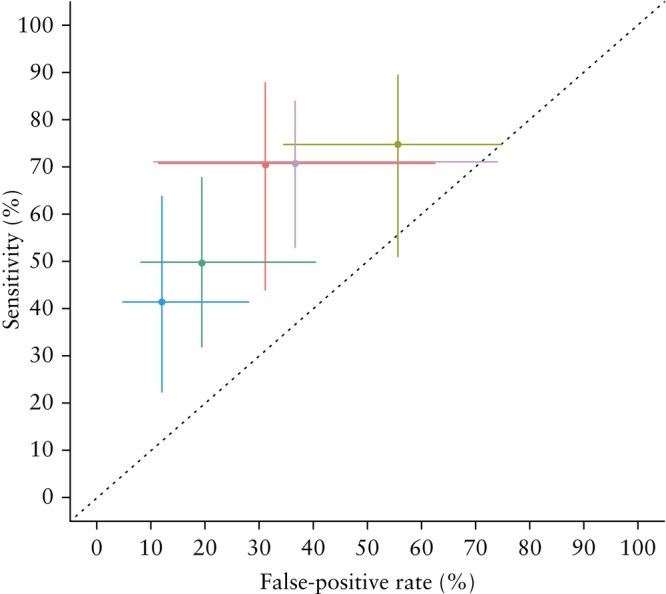
Crosshair plot of pooled sensitivity and pooled false‐positive rate with 95% CIs for phenotypic eligibility criteria used to select cases for prenatal exome sequencing in England (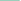), Spain (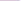), British Columbia (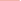), Ontario (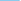) and Greece (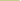).

The performance of the phenotypic eligibility criteria for pES is summarized in Table 2 and Figure 1. Univariate forest plots of sensitivity and specificity for each study and sROC curves for the eligibility criteria of each country/region are shown in Figures S2–S11. All phenotypic criteria performed moderately well, with the best performance seen for the British Columbia criteria, which had a pooled sensitivity of 70.5% (95% CI, 43.7–88.1%), pooled specificity of 68.9% (95% CI, 37.5–89.1%) and AUC of 0.73 (95% CI, 0.58–0.79). The Greek criteria proved the most sensitive, but also had the lowest specificity. Matrices of *P*‐values for the pairwise comparisons between the AUCs, pooled sensitivities and pooled specificities of different eligibility criteria are provided in Tables S6–S8.

## DISCUSSION

This study found that pES is offered in over 80% of countries surveyed, most of which perform pES on a case‐by‐case basis according to the fetal phenotype and the associated likelihood of an underlying monogenic condition. Existing international phenotypic criteria for case selection perform moderately well in detecting causative pathogenic or likely pathogenic variants in an unselected fetal cohort, with marginally better performance achieved by the British Columbia criteria.

The NHS R21 phenotypic eligibility criteria for pES have an incremental diagnostic yield of nearly one‐third over CMA/karyotyping, with the highest yields demonstrated for large bilateral echogenic kidneys with normal bladder, NIHF and isolated SLB, which is consistent with recent evidence^4^, ^5^, ^26^, ^27^. Subsequent updates to gene panels for R21 have progressively optimized the diagnostic yield. The Canadian phenotypic criteria performed best in terms of AUC. Both regions in Canada provided highly specific phenotypic criteria, particularly British Columbia, which included phenotypic features such as radial ray defects and ectrodactyly, both known to have a strong association with genetic disease. This differs from the other countries/regions, where inclusion criteria were based predominantly on published systematic reviews, the limitations of which have already been highlighted^28^. Although there was no significant difference in the diagnostic yield as the number of NHS R21 criteria met increased, it is possible that our study was underpowered to detect this difference.

It is debatable whether pES has replaced the need for the specialism of dysmorphology. Our observation that the criteria based primarily on dysmorphological features associated with a *Gestalt* diagnosis performed best suggests that dysmorphology remains foundational in making a unifying genetic diagnosis and is complementary to emerging next‐generation sequencing technologies. Ideally, such technologies should remain in the hands of dysmorphologists. This is the case in countries that lack predefined phenotypic criteria, where the decision to perform pES is often taken solely by a panel of experts and their perception of how likely the pattern of anomalies is to be representative of a monogenic condition^29^. When considering eligibility criteria, one must establish the primary objective of the testing program: to make the greatest number of diagnoses (higher sensitivity) or to optimize cost‐savings and minimize VUS by limiting false positives (higher specificity). Ideally, to optimize sensitivity, any fetus with an anomaly should undergo pES; however, the economic and ethical implications of this policy must be considered and a balance reached. This is pertinent to the NHS, a publicly funded healthcare system, where the recent findings from the Optimizing Exome Prenatal Sequencing Services (EXPRESS) study are informing practice and decision‐making^30^. As the cost of sequencing and proportion of VUS reduce, it may become feasible to implement broader inclusion criteria.

The use of a fetal anomaly panel rather than a gene‐agnostic approach for analysis has two major advantages. First, it expedites testing because of the reduced number of variants for analysis; and second, it reduces the number of incidental findings. It does, however, result inevitably in the risk of missing pathogenic variants because of the relevant genes being absent from the panel. Novel genotype–phenotype correlations are reported regularly and, therefore, where a panel approach is applied, it is vital that there is a robust and regular gene curation protocol in place. With the vast number of new publications, this can prove to be time‐consuming and resource‐intensive, unless it is possible to automate some aspects of the process. However, it is worth noting that, where a gene is missing from a panel through lack of evidence, in many cases, there is likely to be insufficient evidence to classify a variant as pathogenic or likely pathogenic and hence report it in a prenatal setting. It is therefore worth considering, if possible, implementing a gene‐agnostic reanalysis (or updated panel reanalysis) protocol. In addition, subsequent pregnancies with similar ultrasound findings to the index pregnancy should not be excluded from testing because of the lack of a pathogenic variant discovered in the first pregnancy, particularly if an updated gene panel is in use.

A strength of the present study is that it is, to our knowledge, one of the first studies to assess international approaches to case selection for pES, with responses from approximately one‐third of countries internationally. In addition, phenotypic criteria were applied to an unselected cohort of structurally abnormal fetuses to minimize bias. However, several limitations should be acknowledged. First, the phenotypic descriptions applied within the studies comprising the virtual historical cohort may not have been accurate or representative of phenotypic evolution. Second, the international phenotypic criteria applied to the virtual historical cohort represent a crude list that is subjective and may be extended on a case‐by‐case basis, as stated in each set of guidelines. Third, responses to the survey were conflicting in around half of cases, suggesting that practice differs regionally within countries, or that there is a discrepancy in knowledge regarding the availability of testing or case selection. Fourth, the virtual historical cohort was based on a systematic review performed in 2022. We did not update this cohort because of the challenge of identifying studies including fetuses that are ‘unselected’ for pES. In many studies, the reason for performing pES is inherently biased by the wealth of data regarding the diagnostic yield for selected fetal anomalies. Finally, subanalysis of sequential NHS gene panels according to phenotypic subgroup was not feasible because of the low number of cases in certain subgroups.

In conclusion, in the majority of countries for which there was a survey response, pES was offered on a case‐by‐case basis, according to fetal phenotype and the likelihood of an underlying monogenic condition. Existing international phenotypic criteria evaluated herein performed modestly. Although yet to be assessed formally, the optimal selection process for pES is likely to be one based on expert dysmorphological opinion. Future considerations for advancing the performance of pES include international collaboration in the form of registries or Delphi consensus around how pES should be and is being delivered, in addition to the development of international guidance for clinicians in this area. Such guidance has been recently commissioned by the International Society of Ultrasound in Obstetrics and Gynecology (ISUOG). In the meantime, countries must individually weigh the benefits of high diagnostic yield achieved by offering pES for any fetal anomaly with the financial cost and burden of VUS and incidental findings.

## Supporting information


**Figure S1** Flowchart showing selection of virtual historical genotype–phenotype cohort.
**Figures S2–S6** Forest plots showing pooled sensitivity and specificity for phenotypic eligibility criteria used to select cases for prenatal exome sequencing in England (Figure S2), Ontario (Figure S3), Greece (Figure S4), British Columbia (Figure S5) and Spain (Figure S6).
**Figures S7–S11** Summary receiver‐operating‐characteristics curves for performance of phenotypic eligibility criteria used to select cases for prenatal exome sequencing in England (Figure S7), Ontario (Figure S8), Greece (Figure S9), British Columbia (Figure S10) and Spain (Figure S11).

**Table S1** Responses from international survey (excluding UK) of eligibility criteria for prenatal exome sequencing (pES)
**Table S2** Characteristics of unselected cohorts of fetuses that underwent exome sequencing included in systematic review of Mellis *et al*.^3^
**Table S3** Characteristics of unselected cohorts of fetuses that underwent prenatal exome sequencing included in present study
**Table S4** Pathogenic and likely pathogenic variants identified in virtual historical cohort and year they were added to NHS R21 ‘green’ gene panel
**Table S5** Genes added sequentially to and genes missing from NHS R21 ‘green’ gene panel
**Tables S6–S8** Matrices of *P*‐values for pairwise comparisons between areas under summary receiver‐operating‐characteristics curves (Table S6), pooled sensitivities (Table S7) and pooled specificities (Table S8) of international phenotypic eligibility criteria for prenatal exome sequencing

**Appendix S1** Fetal phenotypic eligibility criteria for prenatal exome sequencing in Ontario, Canada

## Data Availability

The data that support the findings of this study are available from the corresponding author upon reasonable request.

## References

[1] Drexler KA , Talati AN , Gilmore KL , et al. Association of deep phenotyping with diagnostic yield of prenatal exome sequencing for fetal brain abnormalities. Genet Med. 2023;25(10):100915. 37326029 10.1016/j.gim.2023.100915 PMC10580430

[2] Mone F , Homfray T , Kagan KO , Kilby MD . Enhancement of phenotyping for fetal investigation using next‐generation sequencing. Ultrasound Obstet Gynecol. 2023;62(4):459‐461. 37401773 10.1002/uog.26301

[3] Mellis R , Oprych K , Scotchman E , Hill M , Chitty LS . Diagnostic yield of exome sequencing for prenatal diagnosis of fetal structural anomalies: a systematic review and meta‐analysis. Prenat Diagn. 2022;42(6):662‐685. 35170059 10.1002/pd.6115 PMC9325531

[4] Mone F , Mellis R , Gabriel H , et al. Should we offer prenatal exome sequencing for intrauterine growth restriction or short‐long bones? A systematic review and meta‐analysis. Am J Obstet Gynecol. 2023;228(4):409‐417.e4. 36209938 10.1016/j.ajog.2022.09.045

[5] Mone F , Eberhardt RY , Hurles ME , et al. Fetal hydrops and the Incremental yield of Next‐generation sequencing over standard prenatal Diagnostic testing (FIND) study: prospective cohort study and meta‐analysis. Ultrasound Obstet Gynecol. 2021;58(4):509‐518. 33847422 10.1002/uog.23652 PMC8487902

[6] PanelApp . Genomics England. https://panelapp.genomicsengland.co.uk/ [Accessed 1st December 2024].

[7] Abulí A , Antolín E , Borrell A , et al. Guidelines for NGS procedures applied to prenatal diagnosis by the Spanish Society of Gynecology and Obstetrics and the Spanish Association of Prenatal Diagnosis. J Med Genet. 2024;61(8):727‐733. 38834294 10.1136/jmg-2024-109878

[8] Hellenic Society of Ultrasound in Obstetrics and Gynecology . Guidelines for the application of next‐generation sequencing in prenatal screening [translated]. March 2024. https://www.hellenicultrasound.gr/images/hellenicultrasound/sinedria/NGS‐WES‐2024.pdf [Accessed 1st December 2024]

[9] Mone F , Abu Subieh H , Doyle S , et al. Evolving fetal phenotypes and clinical impact of progressive prenatal exome sequencing pathways: cohort study. Ultrasound Obstet Gynecol. 2022;59(6):723‐730. 34940998 10.1002/uog.24842

[10] National Genomic Test Directory . NHS England. https://www.england.nhs.uk/publication/national‐genomic‐test‐directories/ [Accessed 1st December 2024].

[11] Richards S , Aziz N , Bale S , et al. Standards and guidelines for the interpretation of sequence variants: a joint consensus recommendation of the American College of Medical Genetics and Genomics and the Association for Molecular Pathology. Genet Med. 2015;17(5):405‐424. 25741868 10.1038/gim.2015.30 PMC4544753

[12] Association for Clinical Genomic Science . https://www.acgs.uk.com/media/12533/uk‐practice‐guidelines‐for‐variant‐classification‐v12‐2024.pdf [Accessed 1st December 2025].

[13] Reitsma J , Glas A , Rutjes A , Scholten R , Bossuyt P , Zwinderman A . Bivariate analysis of sensitivity and specificity produces informative summary measures in diagnostic reviews. J Clin Epidemiol. 2005;58:982‐990. 16168343 10.1016/j.jclinepi.2005.02.022

[14] Balduzzi S , Rücker G , Schwarzer . How to perform a meta‐analysis with R: a practical tutorial. Evid Based Ment Health. 2019;22:153‐160. 31563865 10.1136/ebmental-2019-300117 PMC10231495

[15] Doebler P , Holling H . Meta‐analysis of diagnostic accuracy with mada. 2017 https://cran.r‐project.org/web/packages/mada/vignettes/mada.pdf [Accessed 1st December 2024]

[16] Drury S , Williams H , Trump N , et al. Exome sequencing for prenatal diagnosis of fetuses with sonographic abnormalities. Prenat Diagn. 2015;35(10):1010‐1017. 26275891 10.1002/pd.4675

[17] Chen M , Chen J , Wang C , et al. Clinical application of medical exome sequencing for prenatal diagnosis of fetal structural anomalies. Eur J Obstet Gynecol Reprod Biol. 2020;251:119‐124. 32502767 10.1016/j.ejogrb.2020.04.033

[18] Li R , Fu F , Yu Q , et al. Prenatal exome sequencing in fetuses with congenital heart defects. Clin Genet. 2020;98(3):215‐230. 32410215 10.1111/cge.13774

[19] Yang X , Huang LY , Pan M , et al. Exome sequencing improves genetic diagnosis of fetal increased nuchal translucency. Prenat Diagn. 2020;40(11):1426‐1431. 32668055 10.1002/pd.5789

[20] Zhou J , Yang Z , Sun J , et al. Whole genome sequencing in the evaluation of fetal structural anomalies: a parallel test with chromosomal microarray plus whole exome sequencing. Genes (Basel). 2021;12(3):376. 33800913 10.3390/genes12030376 PMC7999180

[21] Xue S , Yan H , Chen J , et al. Genetic examination for fetuses with increased fetal nuchal translucency by genomic technology. Cytogenet Genome Res. 2020;160(2):57‐62. 32036363 10.1159/000506095

[22] Sun H , Yi T , Hao X , et al. Contribution of single‐gene defects to congenital cardiac left‐sided lesions in the prenatal setting. Ultrasound Obstet Gynecol. 2020;56(2):225‐232. 31633846 10.1002/uog.21883

[23] Boissel S , Fallet‐Bianco C , Chitayat D , et al. Genomic study of severe fetal anomalies and discovery of GREB1L mutations in renal agenesis. Genet Med. 2018;20(7):745‐753. 29261186 10.1038/gim.2017.173

[24] Zhou X , Wang Y , Shao B , et al. Molecular diagnostic in fetuses with isolated congenital anomalies of the kidney and urinary tract by whole‐exome sequencing. J Clin Lab Anal. 2020;34(11):e23480. 32779812 10.1002/jcla.23480 PMC7676188

[25] Qiao F , Wang Y , Zhang C , et al. Comprehensive evaluation of genetic variants using chromosomal microarray analysis and exome sequencing in fetuses with congenital heart defect. Ultrasound Obstet Gynecol. 2021;58(3):377‐387. 33142350 10.1002/uog.23532

[26] Allen S , Ayeb C , Castleman J , Kinning E , Cilliers D , Kilby M . Abstract 211: prenatal exome sequencing for pregnancies with fetal anomalies – three‐year experience of diagnoses. BMFMS Abstracts 2024. BJOG. 2025;132:3‐105.

[27] Sonner S , Reilly K , Woolf AS , et al. When should we offer antenatal sequencing for urinary tract malformations? A systematic review, cohort study and meta‐analysis. Prenat Diagn. 2024;44(2):187‐195. 38056891 10.1002/pd.6479

[28] Mone F , Rolnik DL , Sotiriadis A , Martinez‐Portilla RJ , Borrell A . Pitfalls of systematic reviews and meta‐analyses to assess the clinical utility of genomic investigations in prenatal diagnosis. Ultrasound Obstet Gynecol. 2024;64(6):713‐715. 39080921 10.1002/uog.29093

[29] Solomon BD , Adam MP , Fong CT , et al. Perspectives on the future of dysmorphology. Am J Med Genet A. 2023;191(3):659‐671. 36484420 10.1002/ajmg.a.63060 PMC9928773

[30] Walton H , Daniel M , Peter M , et al. Evaluating the implementation of the rapid prenatal exome sequencing service in England. Public Health Genomics. 2025;28(1):34‐52. 39667355 10.1159/000543104

