# Supplementary material for: Performance of international phenotypic criteria for prenatal exome sequencing: systematic review and comparative diagnostic accuracy study using historical individual participant data
Source: Ultrasound Obstet Gynecol. 2025 Jul 8;66(3):282–9. doi: 10.1002/uog.29290 (PMC12401499; doi:10.1002/uog.29290)
Supplement: Supplementary file 1 — Figure S1 Flowchart showing selection of virtual historical genotype–phenotype cohort. Figures S2–S6 Forest plots showing pooled sensitivity and specificity for phenotypic eligibility criteria used to select cases for prenatal exome sequencing in England (Figure S2), Ontario (Figure S3), Greece (Figure S4), British Columbia (Figure S5) and Spain (Figure S6). Figures S7–S11 Summary receiver‐operating‐characteristics curves for performance of phenotypic eligibility criteria used to select cases for prenatal exome sequencing in England (Figure S7), Ontario (Figure S8), Greece (Figure S9), British Columbia (Figure S10) and Spain (Figure S11). [file UOG-66-282-s001.docx]

**Figure S1** Flowchart showing selection of virtual historical genotype–phenotype cohort.

No. of studies identified through Mellis, et al. 2022 SR*: (n=72)

No. of studies for which individual data not sought:

Testing not based on prenatal phenotype (n=2)

Targeted sequencing/clinical exome <1000 genes (n=3)

Reasons for not seeking IPD should be reported

Total within final historical genotype-phenotype cohort

No. of studies included in analysis (n=10)

No. of participants included in analysis (n=1054)

No. for which individual data were not provided Positive/negative phenotypic data not available (n=8)

Reasons for not providing IPD should be stated

No. of studies excluded:

Pre-selected cohorts (n=36)

Selection status not disclosed (n=13)

Number of studies where individual data were provided (n=10)

Number of participants for whom data were provided (n=1096)

Number participants for whom no data were provided (n=42; phenotype not clearly stated)

No. of studies for which individual historical case data were sought (n=23)

No. of studies screened for eligibility

(n=72)

No. of studies after duplicates removed N/A

**Figure S2** Forest plots showing pooled sensitivity and specificity for phenotypic eligibility criteria used to select cases for prenatal exome sequencing in NHS England


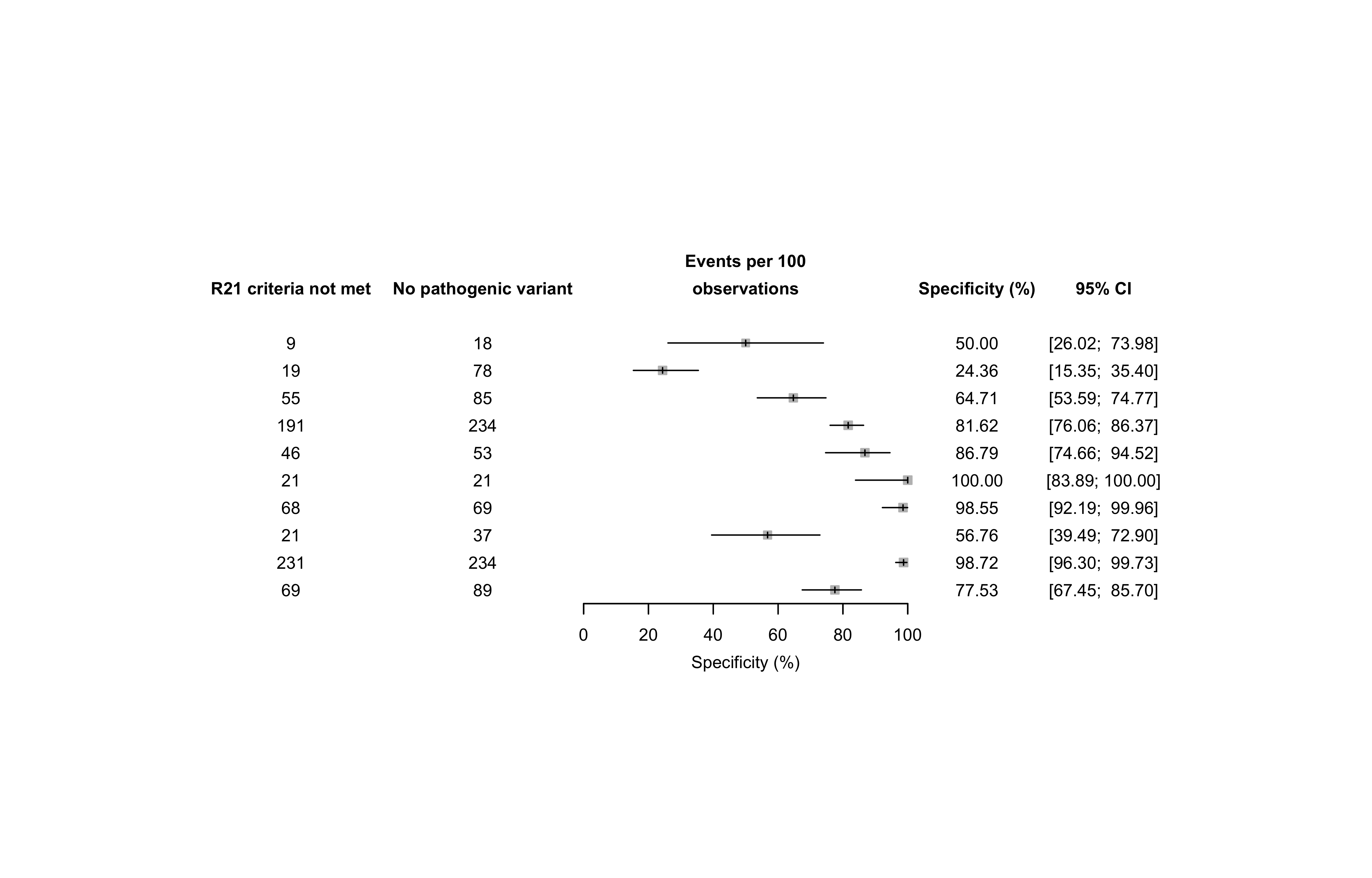

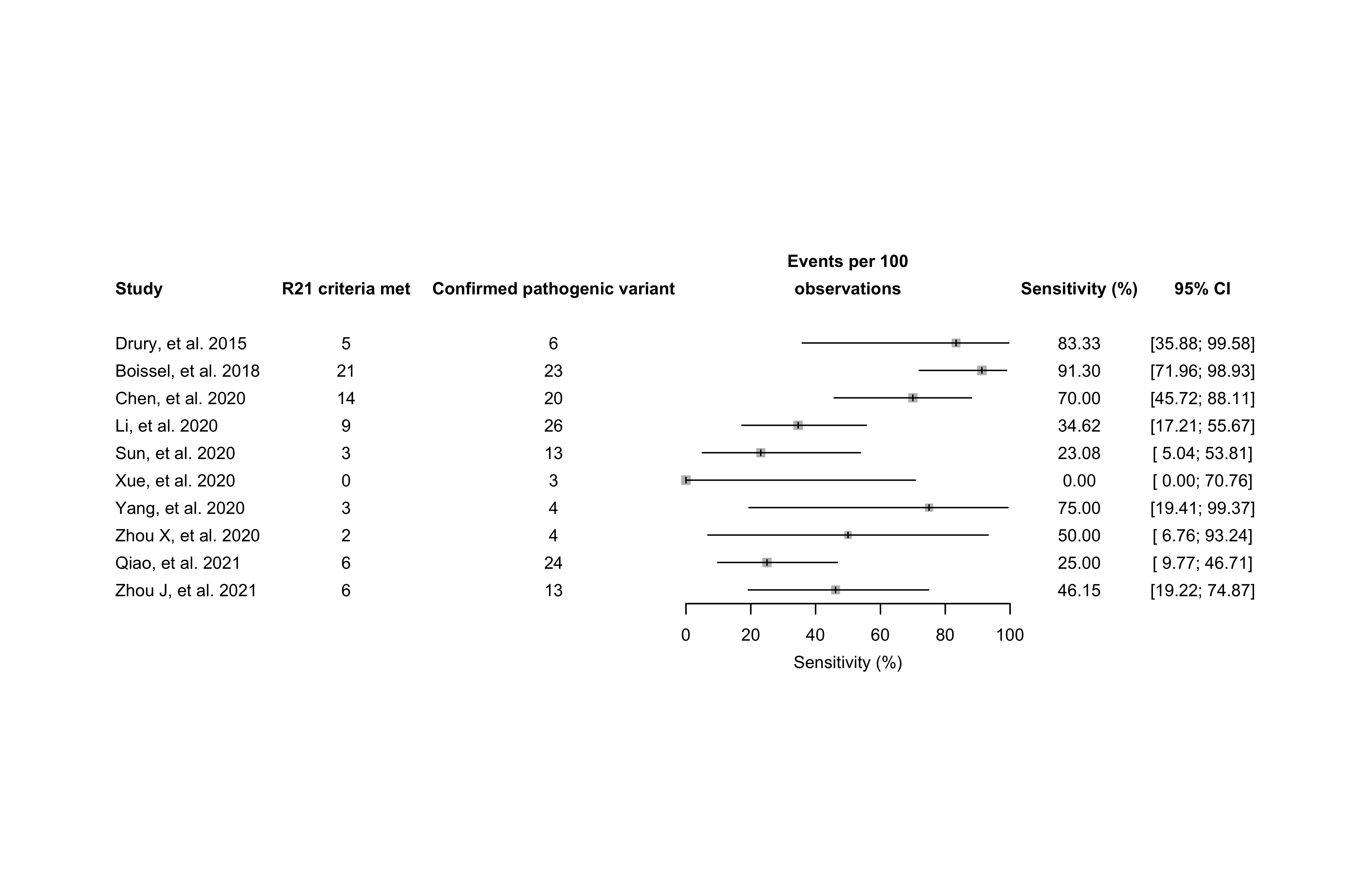


*Univariate analysis of sensitivity and specificity, without pooling: I^2^ Sensitivity: 92.8% (95% CI 88.7% – 95.3%), p < 0.001; I^2^ Specificity: 97.6% (95% CI 96.7% – 98.3%), p < 0.001 [95% CI, 95% confidence interval]

**Figure S3** Forest plots showing pooled sensitivity and specificity for phenotypic eligibility criteria used to select cases for prenatal exome sequencing in Ontario


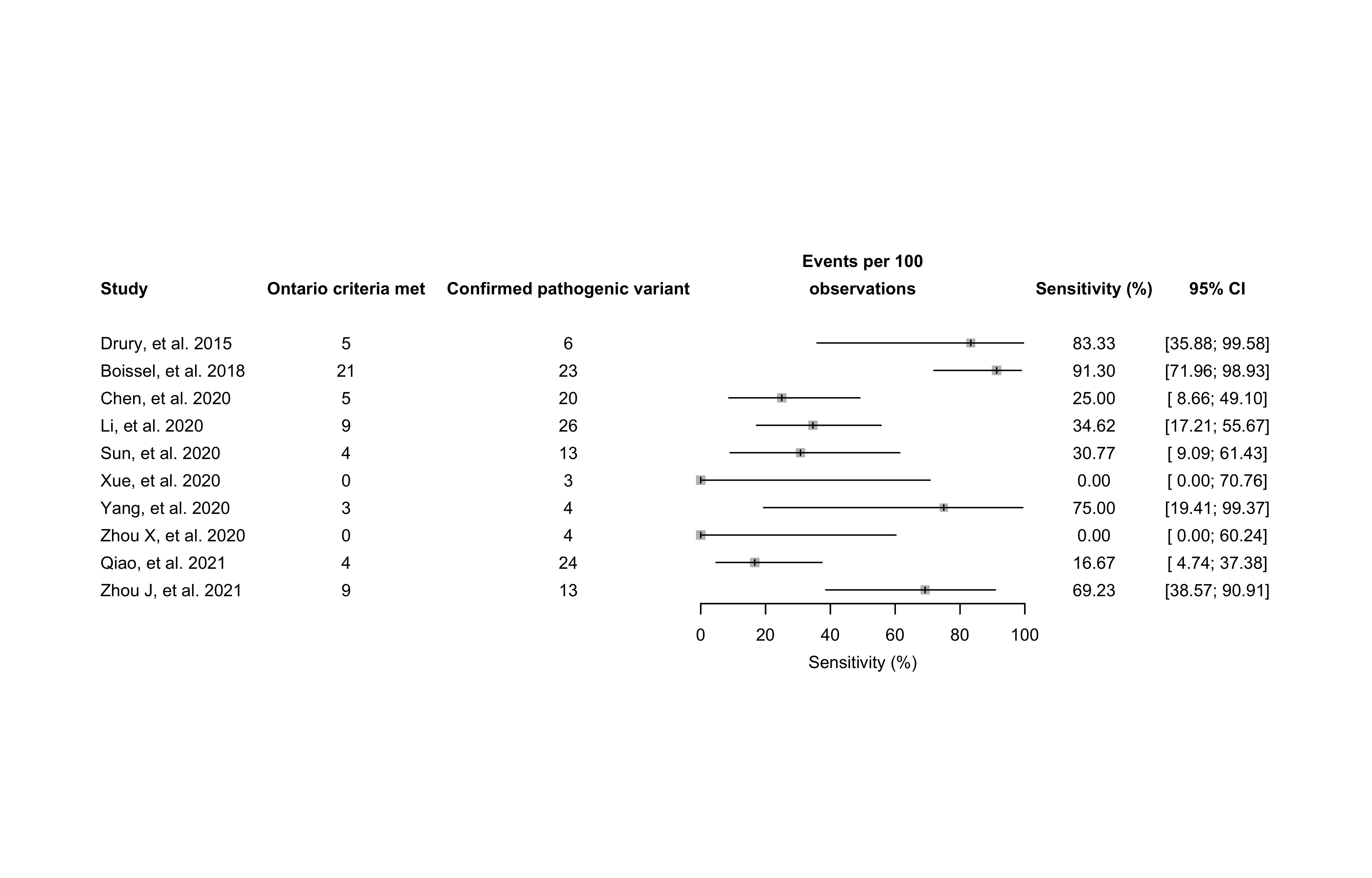

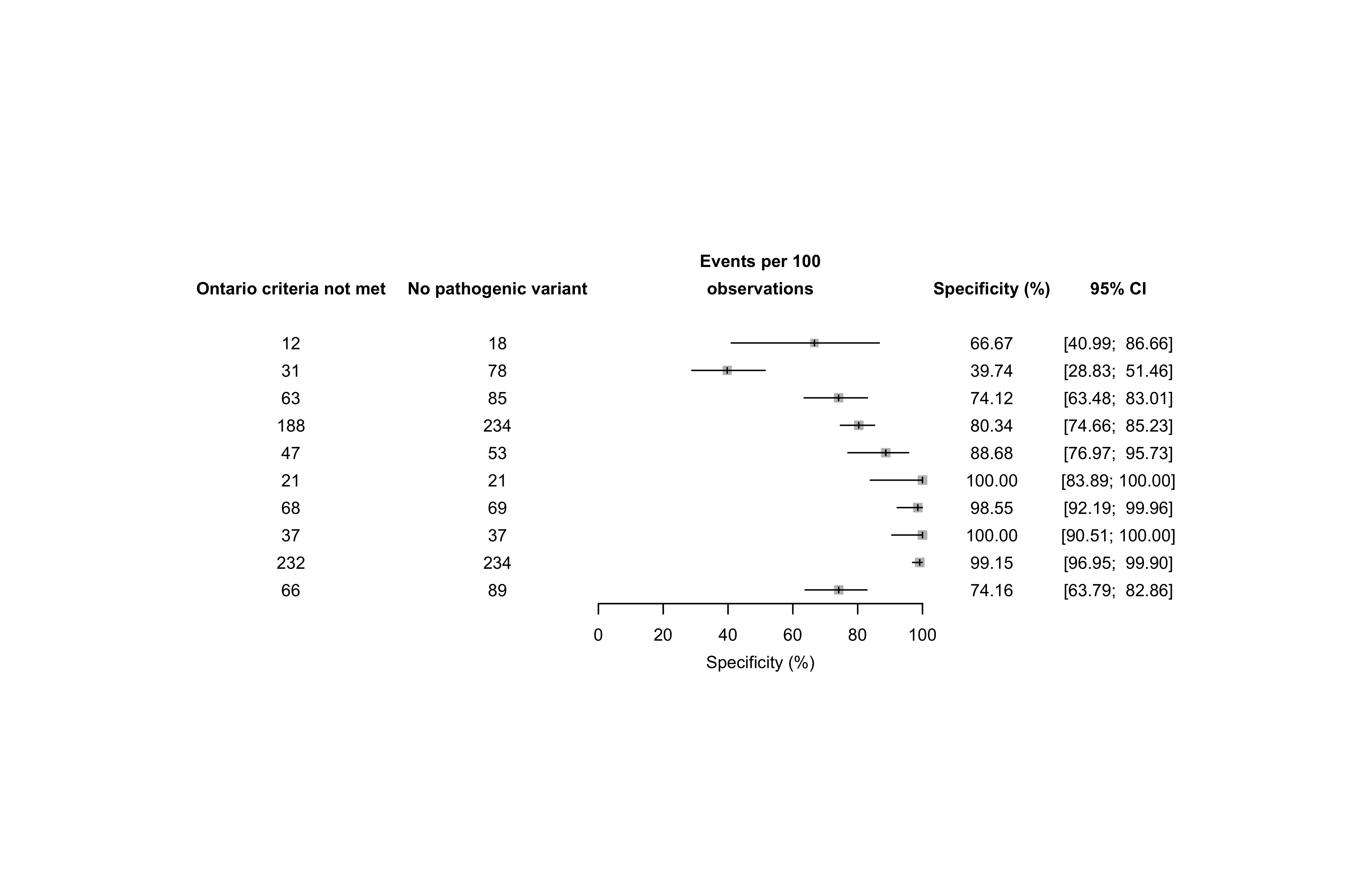


* Univariate analysis of sensitivity and specificity, without pooling; I^2^ Sensitivity: 95.1% (95% CI 92.8% – 96.7%), p < 0.001; I^2^ Specificity: 96.2% (95% CI 94.6% – 97.4%), p < 0.001 [95% CI, 95% confidence interval]

**Figure S4** Forest plots showing pooled sensitivity and specificity for phenotypic eligibility criteria used to select cases for prenatal exome sequencing in Greece


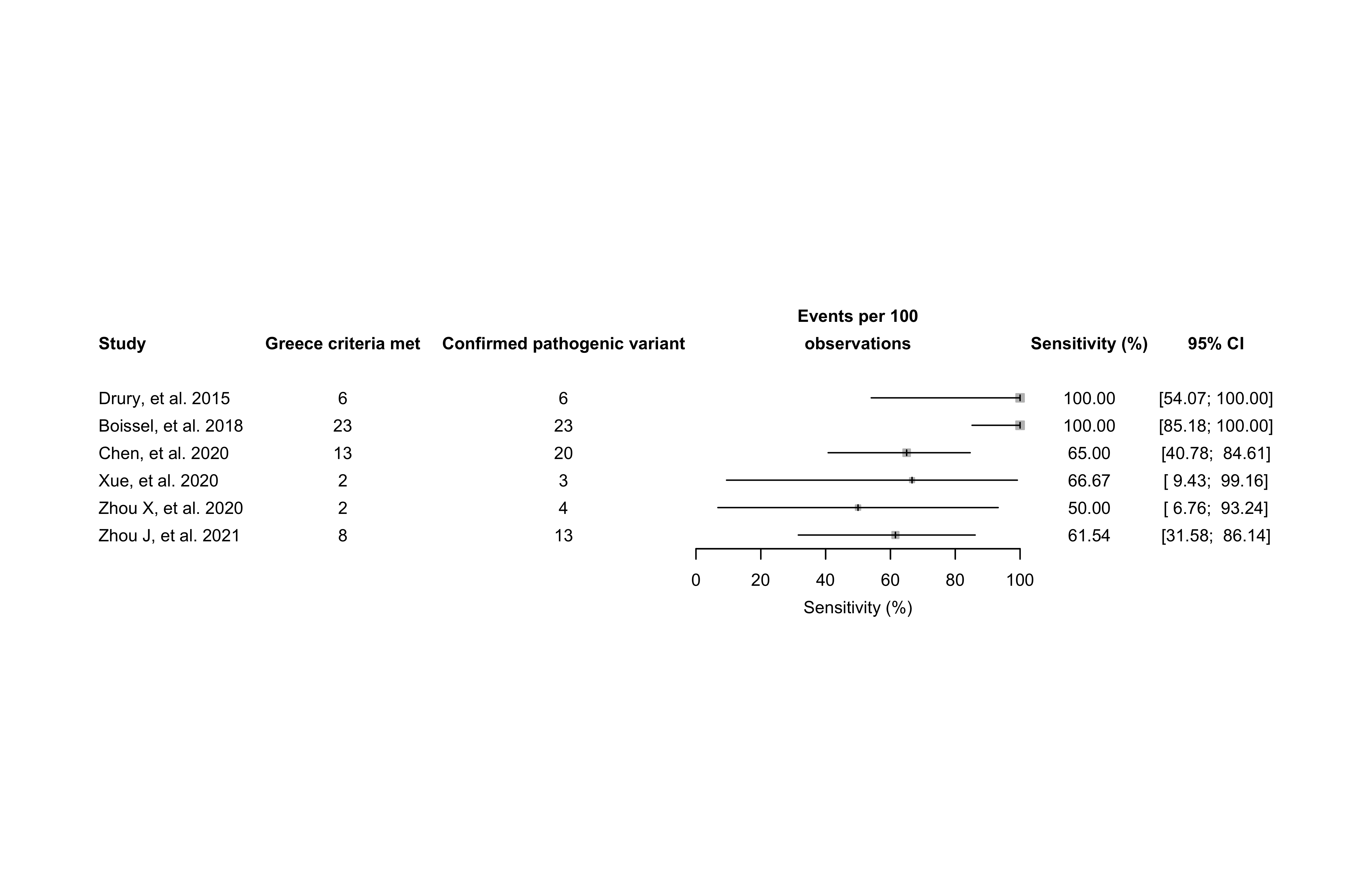

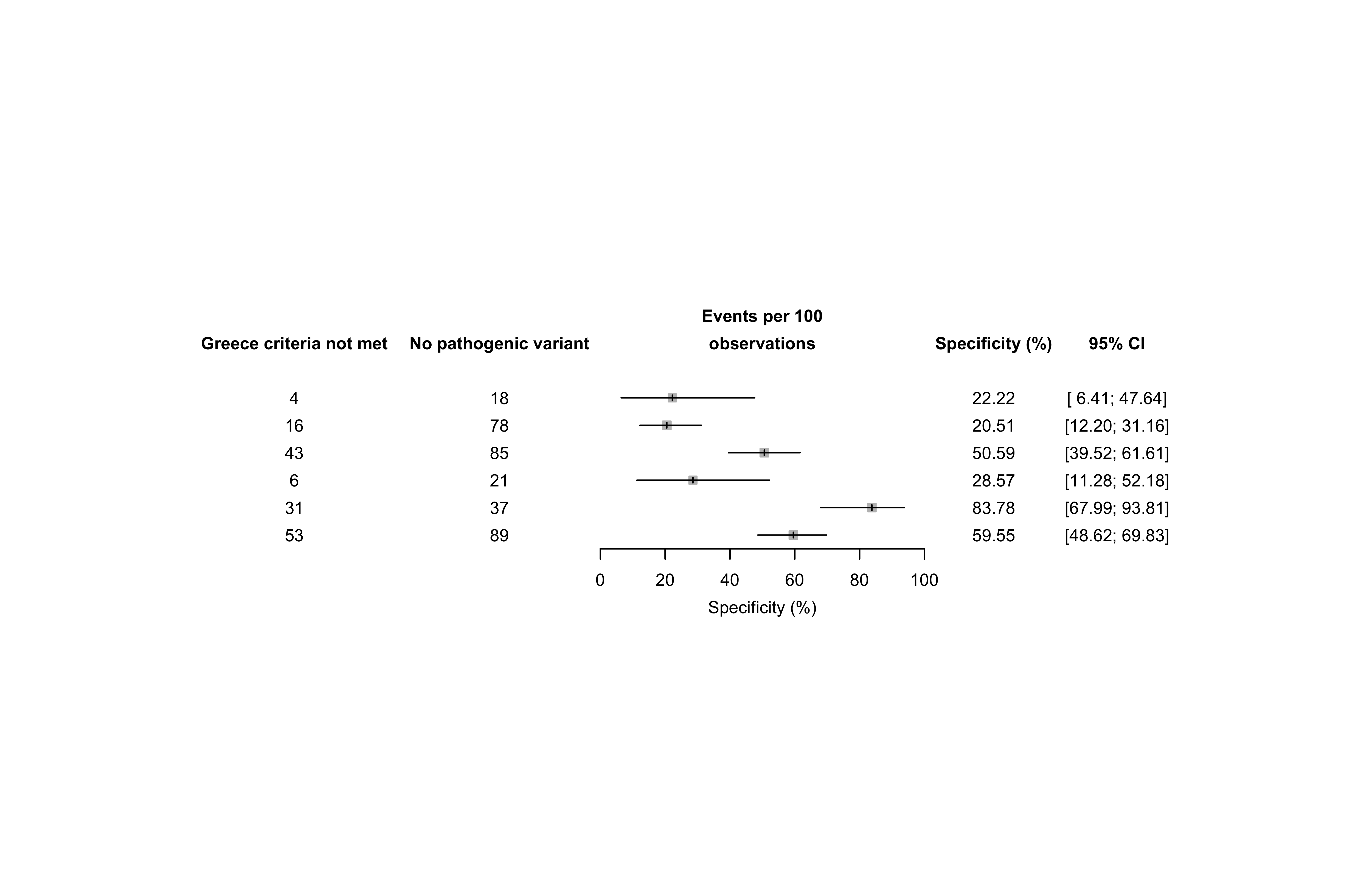


*Univariate analysis of sensitivity and specificity, without pooling; I^2^ Sensitivity: 79.2% (95% CI 54.5% – 90.5%), p < 0.001; I^2^ Specificity: 94.2% (95% CI 90.0% – 96.7%), p < 0.001 [95% CI, 95% confidence interval]

**Figure S5** Forest plots showing pooled sensitivity and specificity for phenotypic eligibility criteria used to select cases for prenatal exome sequencing in British Colombia


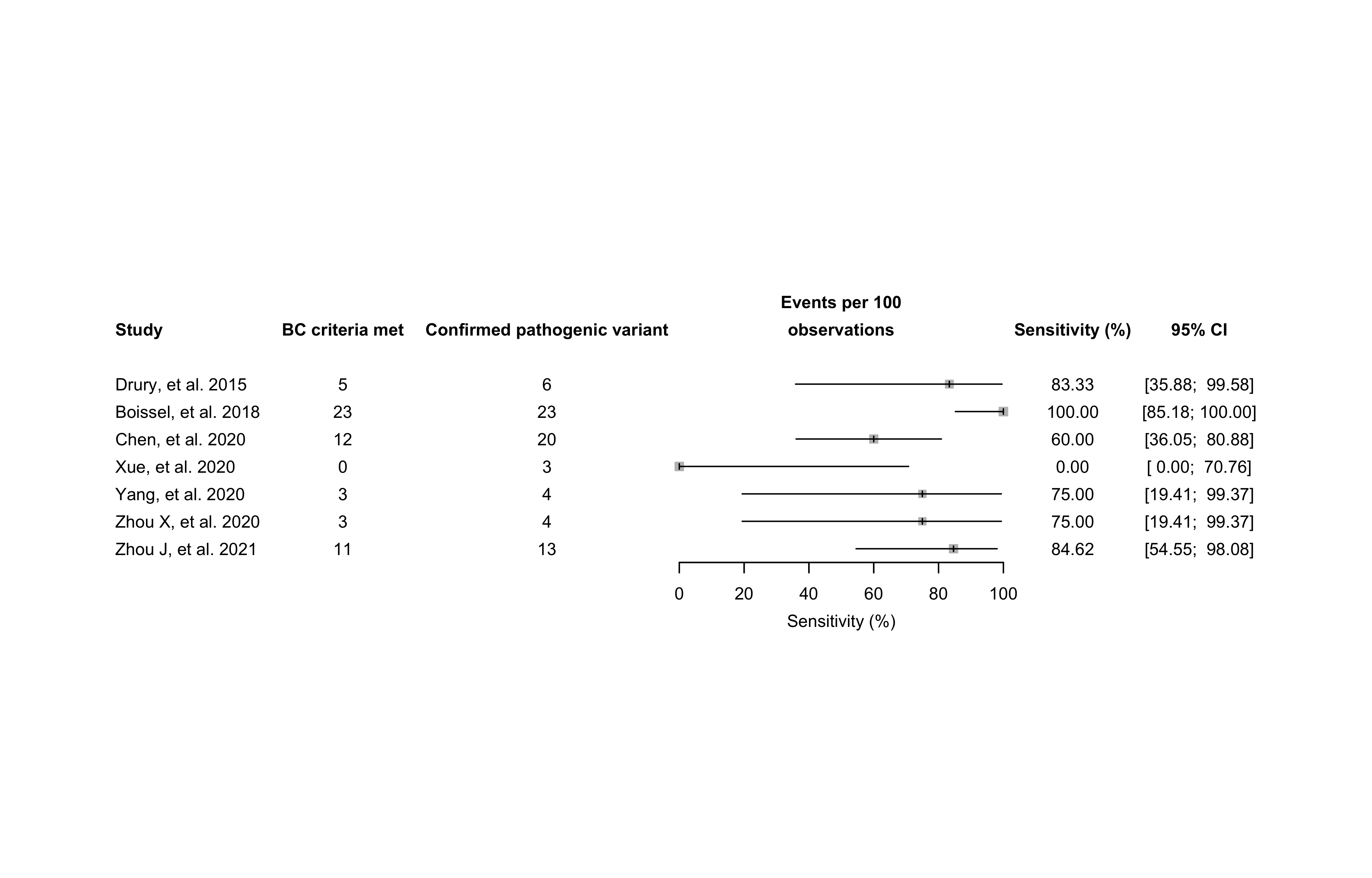

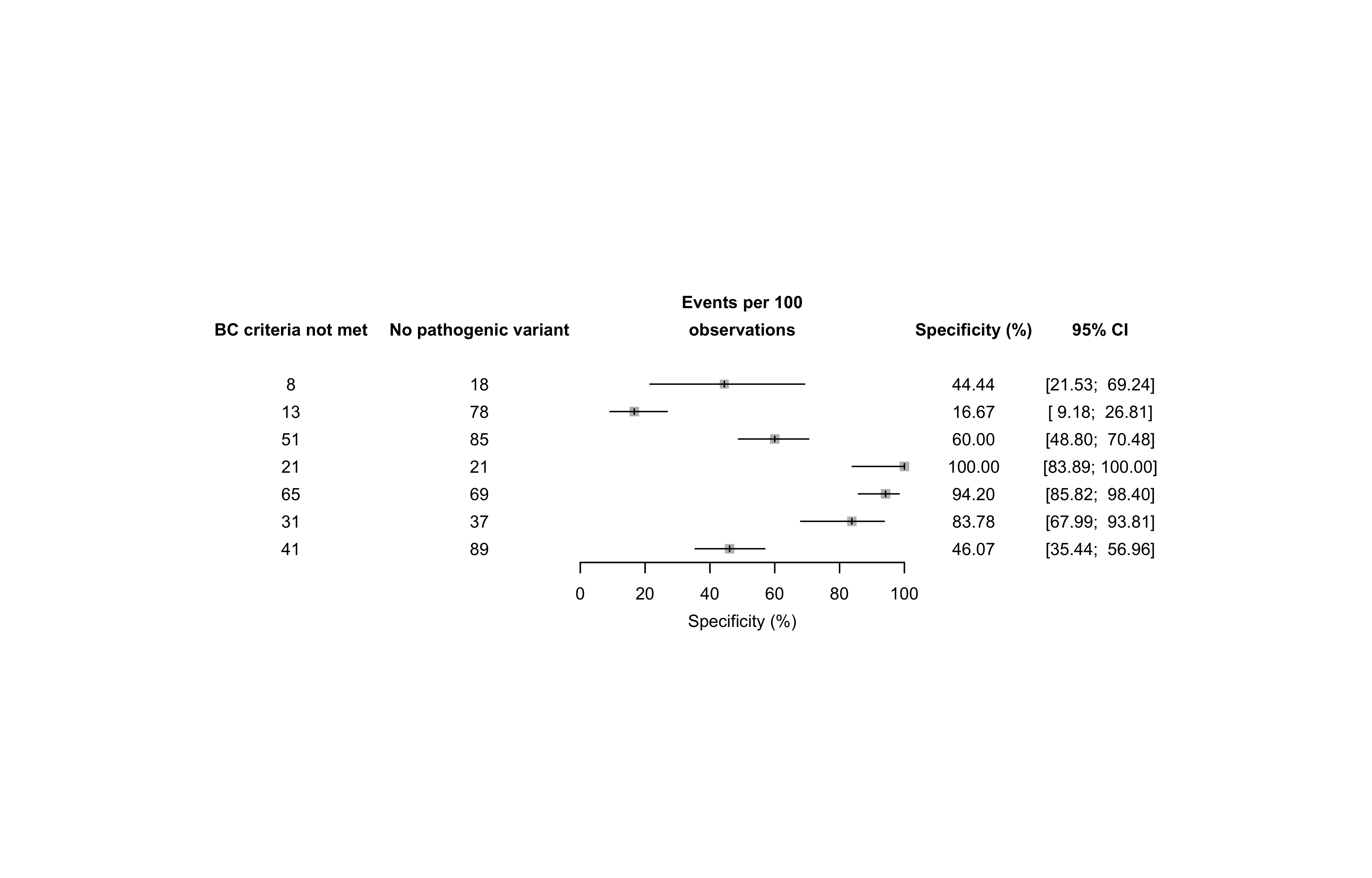


* Univariate analysis of sensitivity and specificity, without pooling; I^2^ Sensitivity: 97.1% (95% CI 95.7% – 98.1%), p < 0.001; I^2^ Specificity: 98.8% (95% CI 98.4% – 99.1%), p < 0.001 [95% CI, 95% confidence interval]

**Figure S6** Forest plots showing pooled sensitivity and specificity for phenotypic eligibility criteria used to select cases for prenatal exome sequencing in Spain


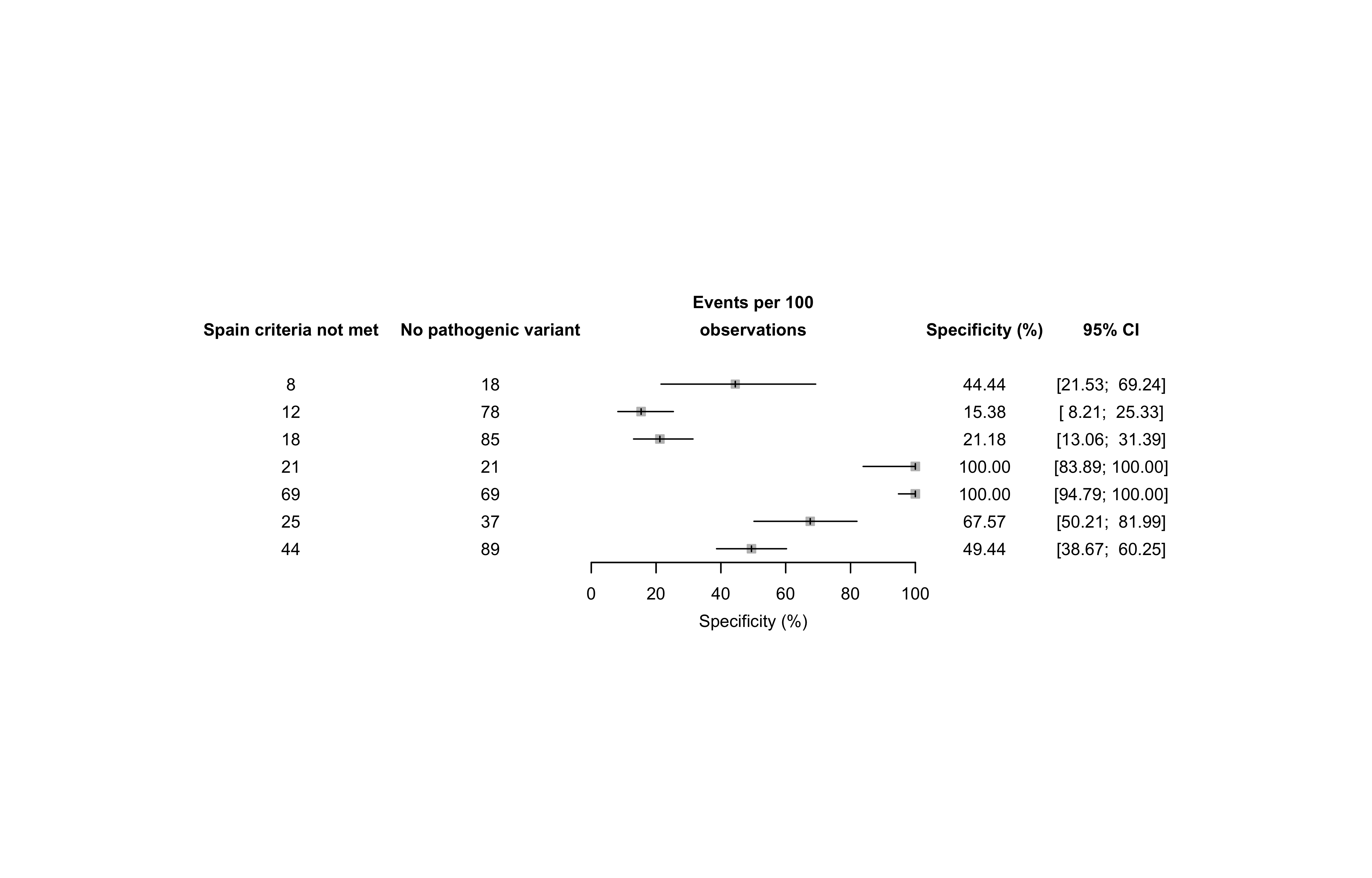

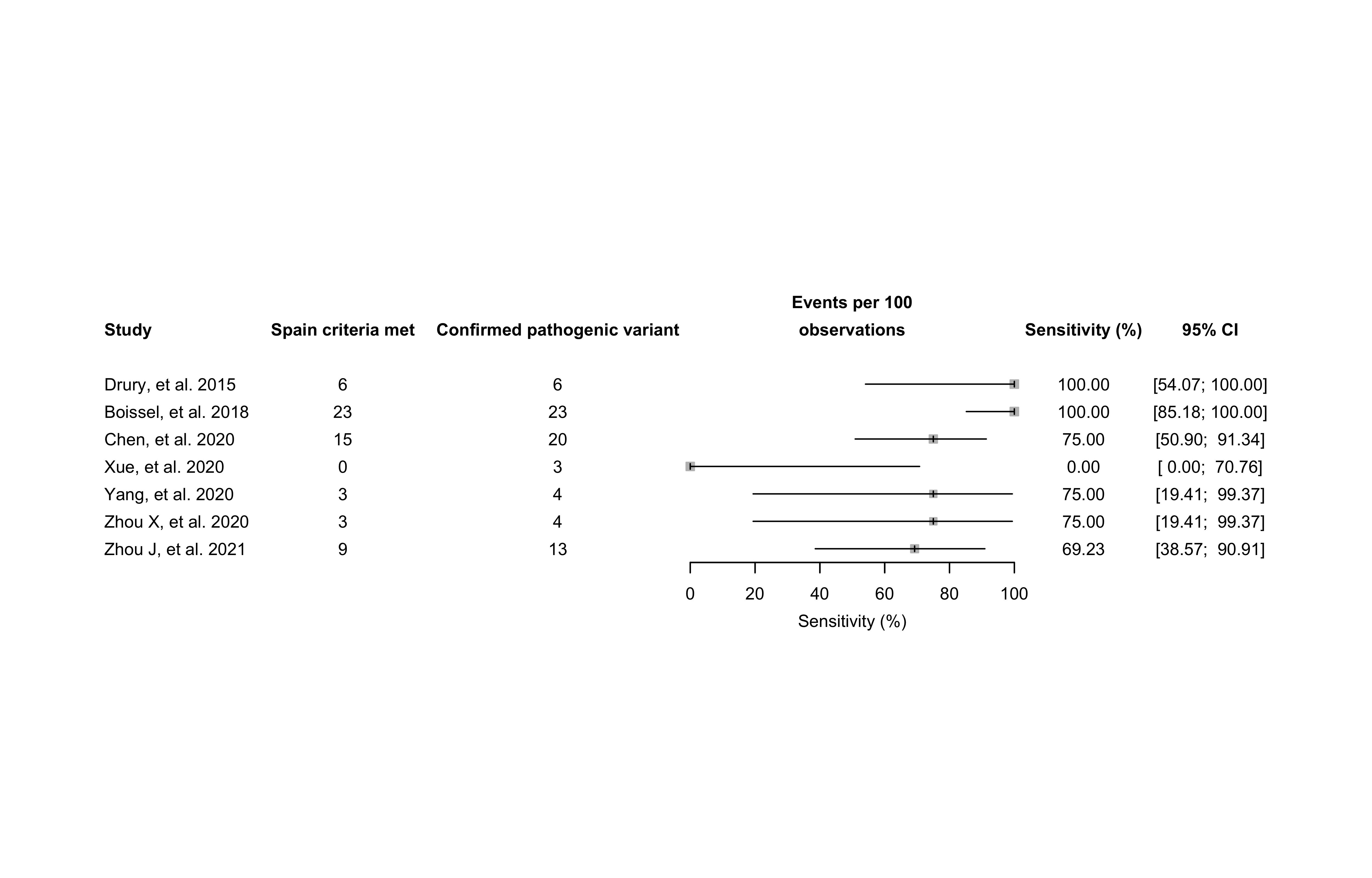


*Univariate analysis of sensitivity and specificity, without pooling; I^2^ Sensitivity: 97.1% (95% CI 95.6% – 98.1%), p < 0.001; I^2^ Specificity: 99.3% (95% CI 99.1% – 99.5%), p < 0.001 [95% CI, 95% confidence interval]

**Figure S7** Summary receiver-operating-characteristics curves for performance of phenotypic eligibility criteria used to select cases for prenatal exome sequencing in NHS England


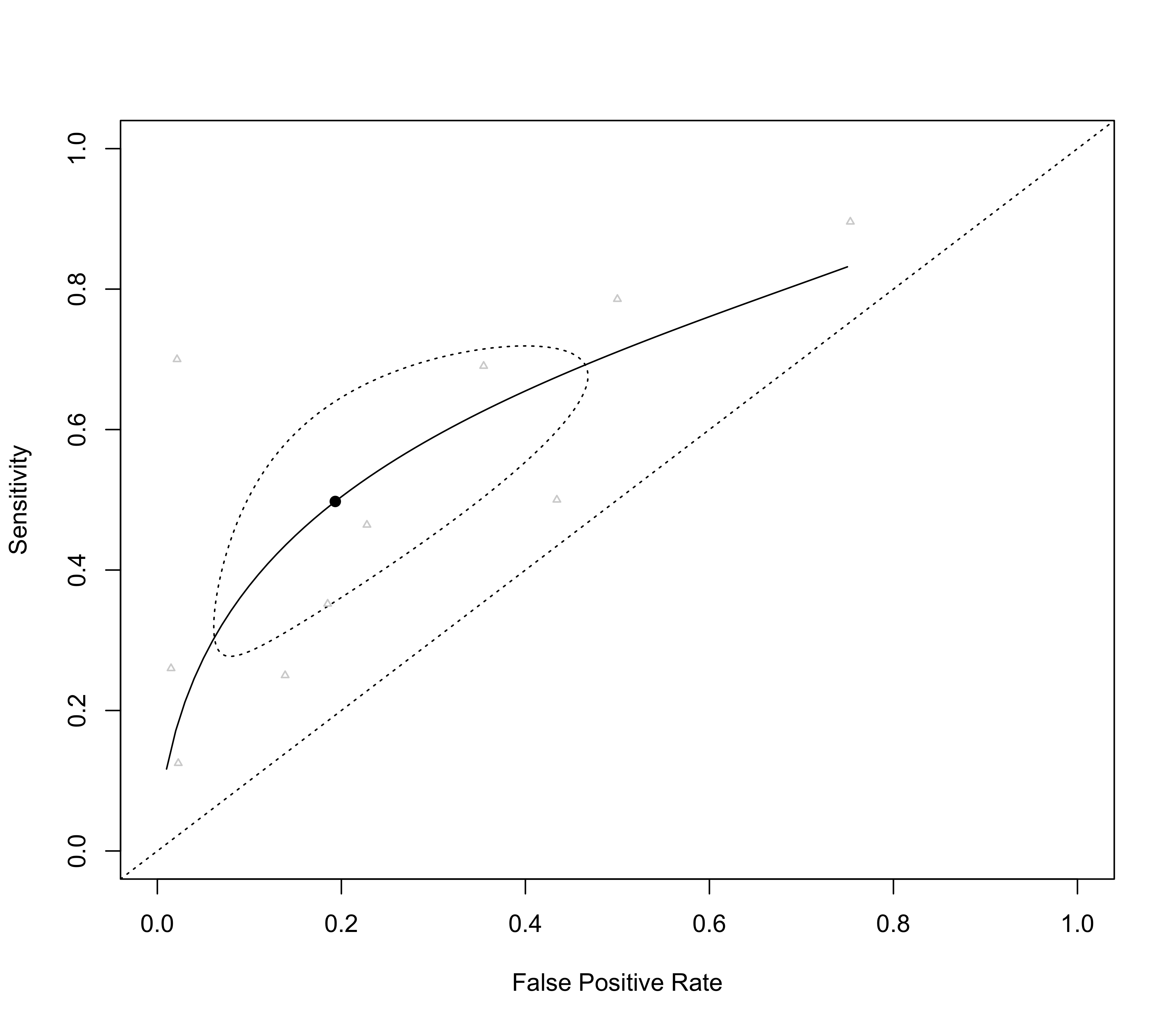


*Bivariate model: Pooled sensitivity: 49.8% (95% CI 31.7% – 67.9%); Pooled false-positive rate (100%-specificity): 19.3% (95% CI 7.8% – 40.5%); Area under the sROC curve 0.660 (95% CI 0.528 – 0.740) (estimated with 5,000 bootstrap samples)

**Figure S8** Summary receiver-operating-characteristics curves for performance of phenotypic eligibility criteria used to select cases for prenatal exome sequencing in Ontario


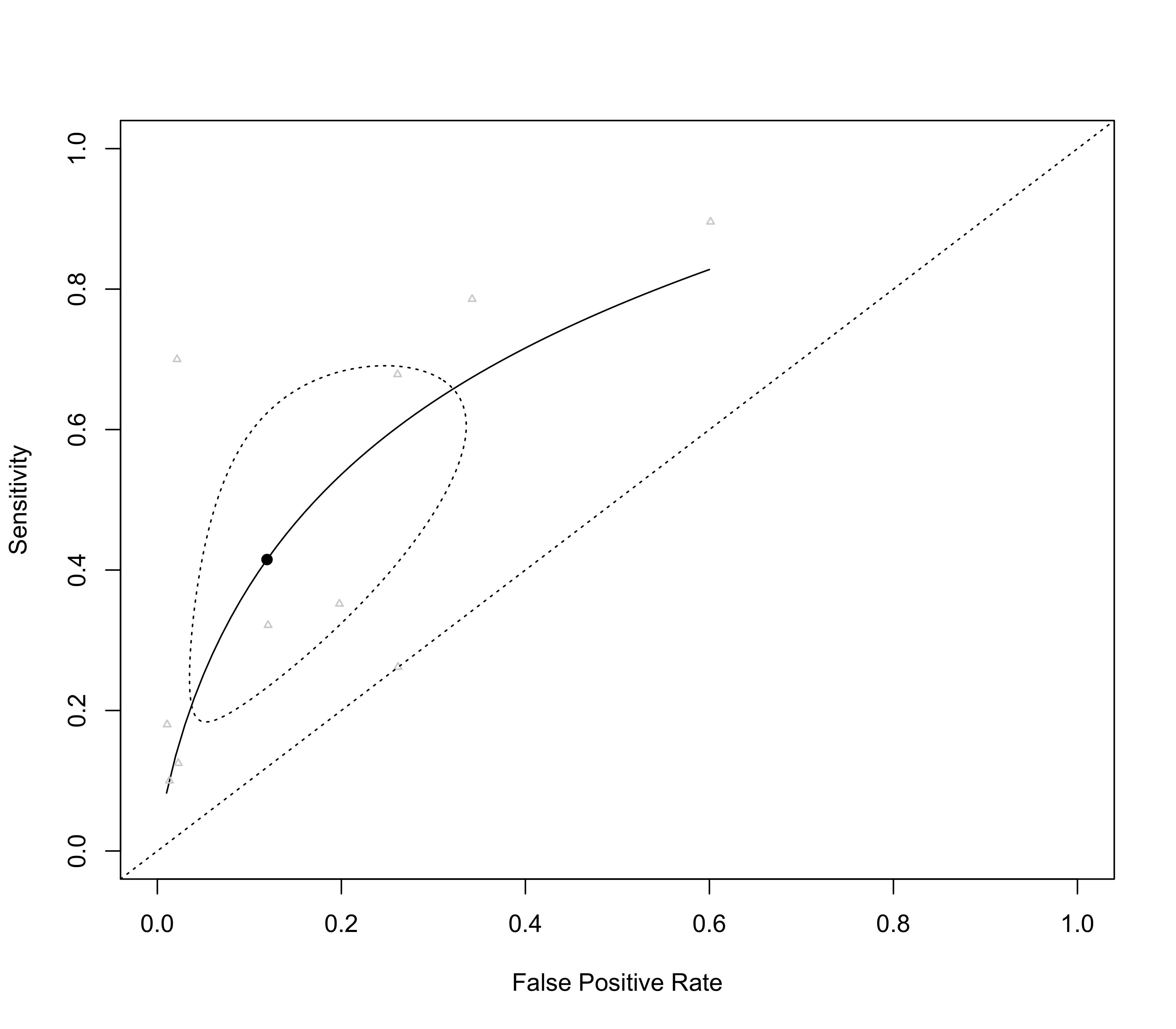


*Bivariate model: Pooled sensitivity: 41.5% (95% CI 22.1% – 64.0%); Pooled false-positive rate (100%-specificity): 11.9% (95% CI 4.5% – 28.0%);

Area under the sROC curve 0.703 (95% CI 0.486 – 0.800) (estimated with 5,000 bootstrap samples)

**Figure S9** Summary receiver-operating-characteristics curves for performance of phenotypic eligibility criteria used to select cases for prenatal exome sequencing in Greece


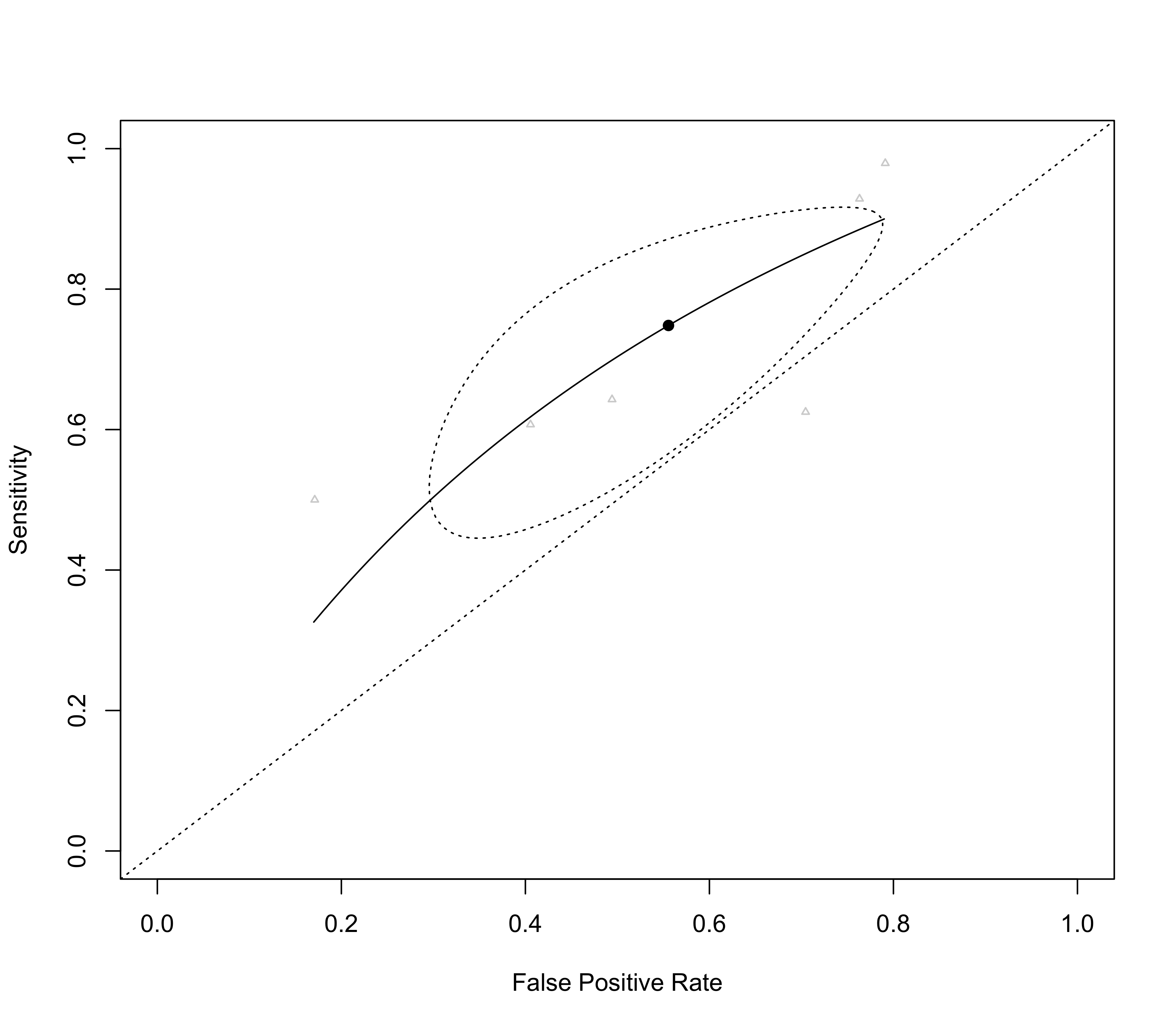


* Bivariate model: Pooled sensitivity: 74.8% (95% CI 51.0% – 89.5%); Pooled false-positive rate (100%-specificity): 55.6% (95% CI 34.3% – 75.0%);

Area under the sROC curve 0.631 (95% CI 0.492 – 0.747) (estimated with 5,000 bootstrap samples

**Figure S10** Summary receiver-operating-characteristics curves for performance of phenotypic eligibility criteria used to select cases for prenatal exome sequencing in British Colombia


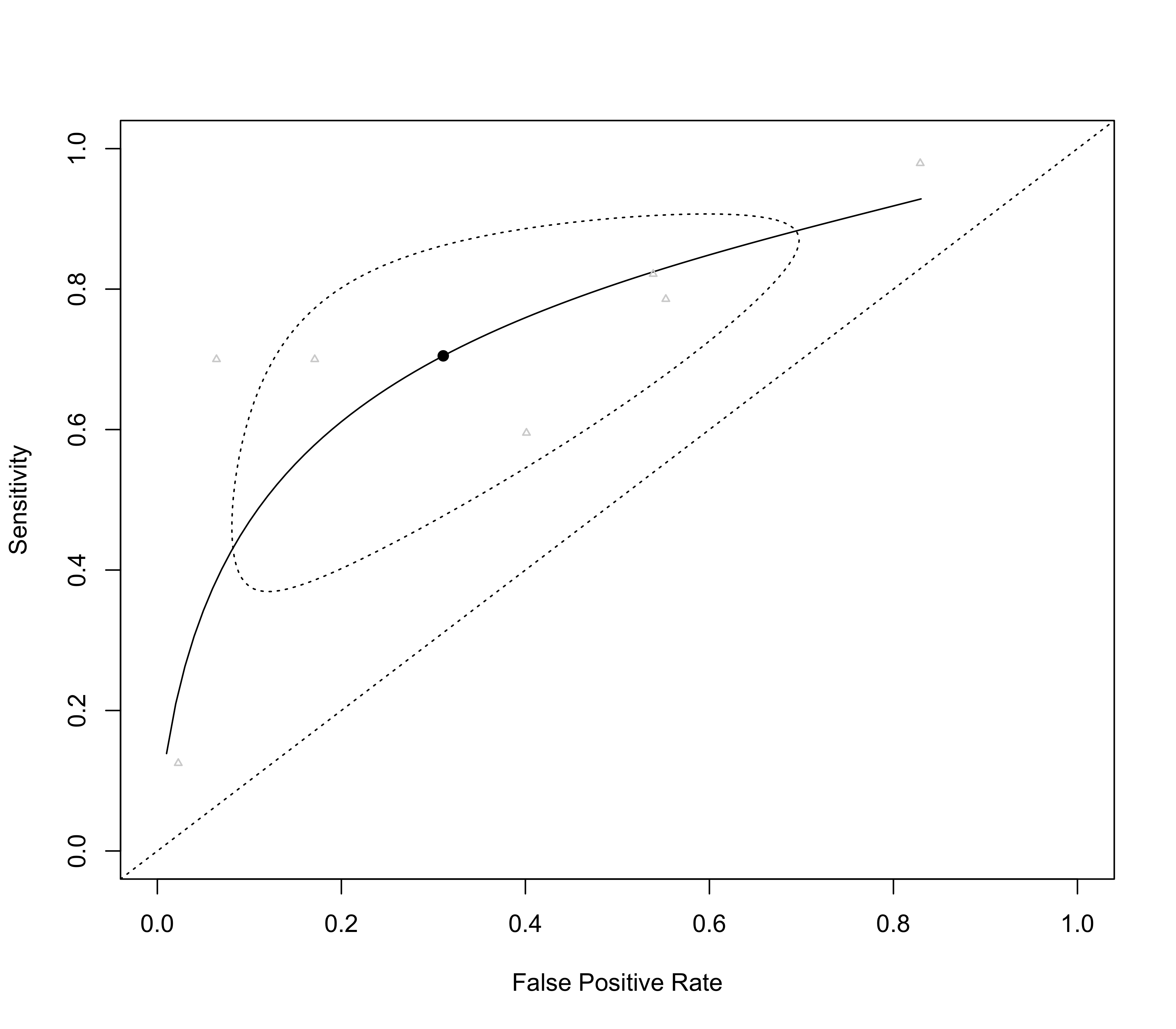


*Bivariate model: Pooled sensitivity: 70.5% (95% CI 43.7% – 88.1%); Pooled false-positive rate (100%-specificity): 31.1% (95% CI 10.9% – 62.5%); Area under the sROC curve 0.730 (95% CI 0.577 – 0.789) (estimated with 5,000 bootstrap samples)

**Figure S11** Summary receiver-operating-characteristics curves for performance of phenotypic eligibility criteria used to select cases for prenatal exome sequencing in Spain


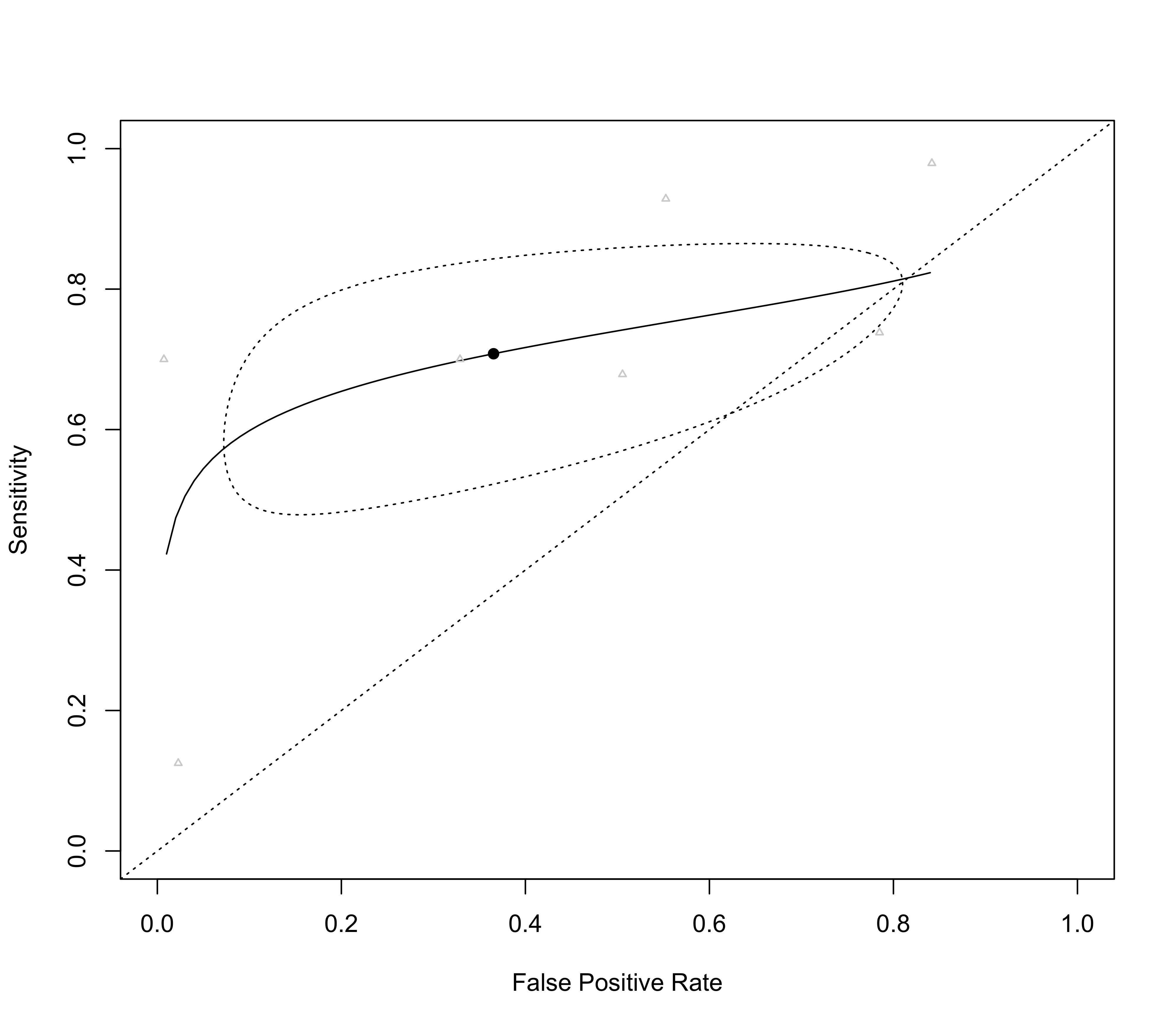


*Bivariate model: Pooled sensitivity: 70.8% (95% CI 52.7% – 84.1%); Pooled false-positive rate (100%-specificity): 36.5% (95% CI 10.4% – 74.1%); Area under the sROC curve 0.709 (95% CI 0.568 – 0.775) (estimated with 5,000 bootstrap samples).
