# Supplementary material for: Performance of international phenotypic criteria for prenatal exome sequencing: systematic review and comparative diagnostic accuracy study using historical individual participant data
Source: Ultrasound Obstet Gynecol. 2025 Jul 8;66(3):282–9. doi: 10.1002/uog.29290 (PMC12401499; doi:10.1002/uog.29290)
Supplement: Supplementary file 2 — Table S1 Responses from international survey (excluding UK) of eligibility criteria for prenatal exome sequencing (pES) Table S2 Characteristics of unselected cohorts of fetuses that underwent exome sequencing included in systematic review of Mellis et al. 3 Table S3 Characteristics of unselected cohorts of fetuses that underwent prenatal exome sequencing included in present study Table S4 Pathogenic and likely pathogenic variants identified in virtual historical cohort and year they were added to NHS R21 ‘green’ gene panel Table S5 Genes added sequentially to and genes missing from NHS R21 ‘green’ gene panel Tables S6–S8 Matrices of P‐values for pairwise comparisons between areas under summary receiver‐operating‐characteristics curves (Table S6), pooled sensitivities (Table S7) and pooled specificities (Table S8) of international phenotypic eligibility criteria for prenatal exome sequencing [file UOG-66-282-s002.docx]

| Country | Response number | pES available:  0=no 1=yes | Selection criteria: 1=case-by case dependent on sequence of anomalies; 2=phenotypic; 3=any fetus can have pES; 4=Not applicable 5= not-specified |
| --- | --- | --- | --- |
| Algeria | 1 | 0 | 4 |
|  | 2 | 0 | 4 |
| Argentina | 1 | 1 | 4 |
|  | 2 | 0 | 5 |
|  | 3 | 1 | 4 |
|  | 4 | 1 | 1 |
|  | 5 | 1 | 3 |
| Australia | 1 | 1 | 5 |
|  | 2 | 1 | 1 |
|  | 3 | 1 | 1 |
|  | 4 | 1 | 1 |
|  | 5 | 1 | 1 |
|  | 6 | 1 | 1 |
| Austria | 1 | 1 | 1 |
|  | 2 | 0 | 4 |
| Azerbaijan | 1 | 1 | 1 |
| Brazil | 1 | 0 | 4 |
|  | 2 | 0 | 4 |
|  | 3 | 1 | 1 |
|  | 4 | 1 | 1 |
|  | 5 | 1 | 1 |
| Bulgaria | 1 | 1 | 1 |
| Canada | 1 | 1 | 1 |
|  | 2 | 1 | 2 |
| Chile | 1 | 1 | 1 |
|  | 2 | 1 | 1 |
|  | 3 | 0 | 4 |
|  | 4 | 1 | 2 |
| China | 1 | 1 | 1 |
|  | 2 | 1 | 1 |
| Columbia | 1 | 0 | 4 |
|  | 2 | 1 | 1 |
|  | 3 | 0 | 4 |
| Croatia | 1 | 1 | 1 |
|  | 2 | 0 | 4 |
| Ecuador | 1 | 0 | 4 |
|  | 2 | 0 | 4 |
| Egypt | 1 | 1 | 3 |
| Estonia | 1 | 1 | 1 |
| Ethiopia | 1 | 0 | 4 |
| Finland | 1 | 1 | 1 |
| Germany | 1 | 1 | 1 |
|  | 2 | 1 | 1 |
|  | 3 | 1 | 1 |
| Greece | 1 | 1 | 5 |
|  | 2 | 1 | 1 |
|  | 3 | 1 | 3 |
| Hong-Kong, China | 1 | 1 | 1 |
| India | 1 | 1 | 1 |
|  | 2 | 1 | 1 |
|  | 3 | 1 | 1 |
|  | 4 | 1 | 1 |
|  | 5 | 1 | 1 |
|  | 6 | 1 | 1 |
|  | 7 | 1 | 1 |
|  | 8 | 1 | 1 |
|  | 9 | 1 | 5 |
|  | 10 | 1 | 1 |
|  | 11 | 1 | 1 |
|  | 12 | 1 | 1 |
|  | 13 | 1 | 1 |
|  | 14 | 1 | 2 |
|  | 15 | 1 | 1 |
|  | 16 | 1 | 1 |
|  | 17 | 1 | 1 |
|  | 18 | 1 | 5 |
|  | 19 | 1 | 1 |
|  | 20 | 1 | 1 |
|  | 21 | 1 | 1 |
|  | 22 | 1 | 1 |
|  | 23 | 1 | 1 |
|  | 24 | 1 | 1 |
|  | 25 | 1 | 5 |
|  | 26 | 1 | 2 |
|  | 27 | 0 | 4 |
|  | 28 | 1 | 5 |
|  | 29 | 1 | 1 |
|  | 30 | 1 | 1 |
|  | 31 | 1 | 1 |
|  | 32 | 1 | 1 |
|  | 33 | 1 | 5 |
|  | 34 | 1 | 1 |
|  | 35 | 1 | 3 |
|  | 36 | 0 | 4 |
| Indonesia | 1 | 0 | 4 |
|  | 2 | 0 | 4 |
|  | 3 | 1 | 1 |
|  | 4 | 1 | 1 |
|  | 5 | 0 | 4 |
|  | 6 | 1 | 1 |
| Iran | 1 | 1 | 1 |
|  | 2 | 1 | 1 |
|  | 3 | 1 | 3 |
| Ireland | 1 | 1 | 1 |
|  | 2 | 1 | 1 |
| Israel | 1 | 1 | 1 |
|  | 2 | 1 | 1 |
|  | 3 | 1 | 1 |
|  | 4 | 1 | 3 |
|  | 5 | 1 | 3 |
| Italy | 1 | 1 | 1 |
|  | 2 | 1 | 2 |
|  | 3 | 1 | 1 |
|  | 4 | 1 | 1 |
|  | 5 | 1 | 1 |
|  | 6 | 1 | 1 |
| Japan | 1 | 1 | 1 |
| Jordan | 1 | 1 | 1 |
|  | 2 | 1 | 1 |
| Kazakstan | 1 | 1 | 2 |
|  | 1 | 1 | 1 |
|  | 2 | 1 | 1 |
|  | 3 | 0 | 4 |
| Kenya | 1 | 1 | 2 |
| Kuwait | 1 | 0 | 4 |
| Lenanon | 1 | 1 | 1 |
| Lithuania | 1 | 1 | 1 |
|  | 2 | 1 | 1 |
| Malaysia | 1 | 0 | 4 |
| North Macedonia | 1 | 0 | 4 |
|  | 2 | 1 | 1 |
|  | 3 | 1 | 5 |
|  | 4 | 0 | 4 |
| Mexico | 1 | 0 | 4 |
|  | 2 | 1 | 1 |
|  | 3 | 1 | 1 |
|  | 4 | 1 | 1 |
|  | 5 | 0 | 4 |
|  | 6 | 0 | 4 |
|  | 7 | 0 | 4 |
|  | 8 | 1 | 1 |
| Namibia | 1 | 0 | 4 |
| Nepal | 1 | 1 | 1 |
| Netherlands | 1 | 1 | 2 |
| Nigeria | 1 | 1 | 1 |
|  | 2 | 0 | 4 |
| Pakistan | 1 | 0 | 4 |
|  | 2 | 0 | 4 |
|  | 3 | 1 | 1 |
|  | 4 | 1 | 1 |
|  | 5 | 0 | 4 |
|  | 6 | 1 | 3 |
|  | 7 | 0 | 4 |
| Panama | 1 | 1 | 1 |
|  | 2 | 1 | 1 |
| Peru | 1 | 1 | 2 |
| Philippines | 1 | 0 | 4 |
| Poland | 1 | 1 | 1 |
|  | 2 | 1 | 1 |
|  | 3 | 1 | 3 |
|  | 4 | 1 | 1 |
| Portugal | 1 | 1 | 1 |
|  | 2 | 1 | 1 |
|  | 3 | 1 | 1 |
|  | 4 | 1 | 1 |
| Romania | 1 | 0 | 4 |
|  | 2 | 1 | 1 |
|  | 3 | 1 | 1 |
|  | 4 | 1 | 1 |
|  | 5 | 0 | 4 |
|  | 6 | 0 | 4 |
|  | 7 | 0 | 4 |
|  | 8 | 1 | 1 |
|  | 9 | 1 | 1 |
| Serbia | 1 | 1 | 1 |
| South Africa | 1 | 1 | 1 |
|  | 2 | 0 | 4 |
|  | 3 | 1 | 2 |
|  | 4 | 1 | 1 |
| Spain | 1 | 1 | 1 |
|  | 2 | 1 | 2 |
|  | 3 | 1 | 2 |
|  | 4 | 1 | 1 |
| Sudan | 1 | 1 | 1 |
| Sweden | 1 | 1 | 1 |
|  | 2 | 1 | 1 |
| Switzerland | 1 | 1 | 1 |
|  | 2 | 1 | 5 |
| Taiwan | 1 | 1 | 3 |
| Thailand | 1 | 1 | 1 |
| Turkey | 1 | 1 | 1 |
|  | 2 | 1 | 1 |
|  | 3 | 1 | 1 |
|  | 4 | 1 | 1 |
|  | 5 | 0 | 4 |
|  | 6 | 1 | 1 |
|  | 7 | 1 | 2 |
|  | 8 | 1 | 1 |
|  | 9 | 1 | 3 |
|  | 10 | 1 | 5 |
| Uganda | 1 | 0 | 4 |
|  | 2 | 1 | 1 |
| Ukraine | 1 | 1 | 1 |
|  | 2 | 1 | 2 |
| United Arab Emirates | 1 | 1 | 1 |
|  | 2 | 1 | 1 |
|  | 3 | 1 | 1 |
|  | 4 | 1 | 5 |
|  | 5 | 1 | 5 |
|  | 6 | 0 | 4 |
| USA | 1 | 1 | 1 |
|  | 2 | 1 | 1 |
|  | 3 | 1 | 5 |
|  | 4 | 1 | 3 |
| Uzbekistan | 1 | 0 | 4 |
| Vietnam | 1 | 1 | 1 |
|  | 2 | 1 | 1 |
|  | 3 | 1 | 1 |
|  | 4 | 1 | 1 |
|  | 5 | 1 | 1 |
|  | 6 | 1 | 1 |
|  | 7 | 1 | 1 |
|  | 8 | 1 | 1 |
|  | 9 | 1 | 1 |

**Table S1** Responses from international survey (excluding UK) of eligibility criteria for prenatal exome sequencing (pES)

|  | Prenatal phenotype described and sequencing based on this | Positive and negative phenotypes reported | WES or CES>1000 genes | Included |
| --- | --- | --- | --- | --- |
| Aarabi, et al. 2018^1^ | Yes | Yes | No | No |
| Boissel, et al. 2018^2^ | Yes | Yes | Yes | Yes |
| Carss, et al. 2014^3^ | Yes | No | Yes | No |
| Chen, et al. 2020^4^ | Yes | Yes | Yes | Yes |
| Choy, et al. 2019^5^ | Yes | Yes | No | No |
| Drury, et al. 2015^6^ | Yes | Yes | Yes | Yes |
| Fu, et al. 2018^7^ | Yes | No | Yes | No |
| Heide, et al. 2020^8^ | Yes | Yes | No | No |
| Lefebvre, et al. 2010^9^ | No | No | Yes | No |
| Lei, et al. 2020^10^ | Yes | No | Yes | No |
| Lei, et al. 2021^11^ | Yes | No | Yes | No |
| Leung, et al. 2018^12^ | Yes | No | Yes | No |
| Li R, et al. 2020^13^ | Yes | Yes | Yes | Yes |
| Lord, et al. 2019^14^ | Yes | No | Yes | No |
| Petrovski, et al. 2019^15^ | Yes | No | Yes | No |
| Qi, et al. 2020^16^ | Yes | No | Yes | No |
| Qiao, et al. 2021^17^ | Yes | Yes | Yes | Yes |
| Quinlan-Jones, et al.2019^18^ | No | Yes | Yes | No |
| Sun, et al. 2020^19^ | Yes | Yes | Yes | Yes |
| Xue, et al. 2020^20^ | Yes | Yes | Yes | Yes |
| Yang, et al. 2020^21^ | Yes | Yes | Yes | Yes |
| Zhou J, et al. 2021^22^ | Yes | Yes | Yes | Yes |
| Zhou X, et al. 2020^23^ | Yes | Yes | Yes | Yes |

**Table S2** Characteristics of unselected cohorts of fetuses that underwent exome sequencing included in systematic review of Mellis *et al.*^24^

[CES, clinical exome sequencing; WES, whole exome sequencing]

|  | Total cases study | Cases included n (%) | Inclusion criteria for study | Sequencing approach |
| --- | --- | --- | --- | --- |
| Boissel, et al. 2018^2^ | 101 | 101 (100) | Fetuses or stillborns with severe anomalies | Solo or Trio WES |
| Chen, et al. 2020^4^ | 105 | 105 (100) | Fetuses with structural anomalies including isolated increased NT >3.5 mm | Trio CES 4000 genes |
| Drury, et al. 2015^6^ | 24 | 24  (100) | Fetuses with structural anomalies and/or increased NT (>3.5mm) | Solo or trio WES |
| Li R, et al. 2020^13^ | 260 | 260 (100) | Fetuses with congenital heart defects +/- other structural anomalies | Trio WES |
| Qiao, et al. 2021^17^ | 300 | 258  (86) | Fetuses with congenital heart defects +/- other structural anomalies | Solo or Trio WES |
| Sun, et al. 2020^19^ | 66 | 66  (100) | Fetuses with cardiac left sided lesions | Solo or Trio WES |
| Xue, et al. 2020^20^ | 24 | 24  (100) | Fetuses with increased NT and no detectable structural malformations | Trio WES |
| Yang, et al. 2020^21^ | 73 | 73  (100) | Fetuses with isolated first trimester increased NT >3.5mm | Trio CES 4200 genes |
| Zhou J, et al. 2021^22^ | 102 | 102 (100) | Fetuses with structural or growth anomalies identified by US scanning | Trio WES and WGS |
| Zhou X, et al. 2020^23^ | 41 | 41  (100) | Fetuses with isolated renal anomalies | Solo or Trio WES |

**Table S3** Characteristics of unselected cohorts of fetuses that underwent prenatal exome sequencing included in present study

[CES; clinical exome sequencing, NT; nuchal translucency; US; ultrasound, WES; whole exome sequencing]

| *Gene* | Variant | Syndrome | On current R21 gene list Y/N v5 (green) | Date added R21 panel green |
| --- | --- | --- | --- | --- |
| *FLT4* | c.3075G > A p.(Met1025Ile) | Milroy Disease | Y | 2019 |
| *APPL* | c.891C > A p.(Tyr297*) | Hypophosphatasia | Y | 2019 |
| *NF1* | c.5606G > T p.Gly1869Val |  | Y | 2019 |
| *COL2A1* | c.1358G > T p.Gly453Val | Achondrogenesis Type 2 | Y | 2019 |
| *MYH3* | c.2014C > T p.Arg672Cys | Freeman Sheldon | Y | 2019 |
| *C3ORF42* | c. 8167C > T p.(Gln2723*) + c.8628C > T p.(Ser2876Ser) | Oral Facial Digital type IV | Y | 2019 |
| *ATP7B* | c.2972C > T | Arthrogyrpoposis/Wilson disease | N |  |
| *ACTB* | c.209C > T p.Pro70Leu | Baraitser-Winter Syndrome | Y | 2019 |
| *GATA4* | c.1325C>T | No Specific syndrome named | Y | 2019 |
| *KMT2D* | c.5625dupA, p.Asp1876fs | Kabuki Syndrome | Y | 2019 |
| *SON* | c.444_456del | ZTTK syndrome | Y | 2019 |
| *GATA4* | c.221C>A, p.Ala74Asp | VSD1 (OMIM subgroup) | Y | 2019 |
| *CHD7* | c.6104-1G>A | CHARGE syndrome | Y | 2019 |
| *JAG1* | c.2122_2125delCAGT, p.Gln708fs | Alagille Syndrome | Y | 2019 |
| *MED13L* | c.4412_c.4413delTG, p.V1471Gfs* 7 | Neurodevelopmental delay and distinctive facial features with or without cardiac defects | Y | 2022 |
| *PLD1* | c.2447G>C, | Cardiac Valvular Defect, developmental | Y | 2021 |
| *HRAS* | c.488(exon5)_c.507del | Costello Syndrome | Y | 2019 |
| *PIEZO2* | c.2535-1G>A | Marden-Walker Syndrome | Y | 2019 |
| *NIPBL* | c.7082T>G, | Cornelia-de-Lange syndrome 1 | Y | 2019 |
| *FLT4* | c.2102delT, p.Val701fs | CHD multiple types, 7 OMIM subgroup | Y | 2019 |
| *PQBP1* | c.461_462delAG, | Renpenning Syndrome | Y | 2019 |
| *GATA6* | c.832G>T, p.Glu278* | Pancreatic agenesis | Y | 2019 |
| *TAB2* | c.814C>T, p.Gln272* | CHD, non-syndromic, 2 OMIM subgroup | Y | 2019 |
| *TBX5* | c.192G>A,p.Trp64 | Holt-Oram syndrome | Y | 2019 |
| *SOS1* | c.2647G>A,p.Val883Ile | Noonan Syndrome | Y | 2019 |
| *KMT2D* | c.12218C>G,p.Ser4073* | Kabuki syndrome | Y | 2019 |
| *PTPN11* | c.836A>G,p.Tyr279Cys | Noonan Syndrome | Y | 2019 |
| *PIEZ02* | c.7169_7170insCGGG | Marden-Walker Syndrome | Y | 2019 |
| *KRAS* | c.179G>T, p.Gly60Val | Noonan Syndrome | Y | 2019 |
| *KMT2D* | c.15143G>A | Kabuki syndrome | Y | 2019 |
| *CHD7* | c.2839C>T, p.R947X | CHARGE syndrome | Y | 2019 |
| *ISPD* | c.934-1(IVS6)G>A | Muscular dystrophy dystroglycanopathy | Y | 2019 |
| *NOTCH1* |  | Aortic valve disease 1, AD; Adams– Oliver syndrome 5, AD | Y | 2019 |
| *KMT2D* |  | Kabuki Syndrome 1 | Y | 2019 |
| *NOTCH1* |  | Aortic valve disease 1, AD; Adams– Oliver syndrome 5, AD | Y | 2019 |
| *KMT2D* |  | Kabuki Syndrome 1 | Y | 2019 |
| *MYRF* |  | Cardiac–urogenital syndrome, AD | Y | 2019 |
| *KMT2D* |  | Kabuki Syndrome 1 | Y | 2019 |
| *NOTCH1* |  | Aortic valve disease 1, AD; Adams– Oliver syndrome 5, AD | Y | 2019 |
| *KMT2D* |  | Kabuki Syndrome 1 | Y | 2019 |
| *KMT2D* |  | Kabuki Syndrome 1 | Y | 2019 |
| *NOTCH1* |  | Aortic valve disease 1, AD; Adams– Oliver syndrome 5, AD | Y | 2019 |
| *PTPN11* | c.166A>G, p.lle56Val | Noonan syndrome 1 | Y | 2019 |
| *ABL1* | c.431A>G, p.His114Arg | Congenital heart defects and skeletal malformations syndrome | Y | 2021 |
| *MYH7* | c.2135G>A, p.Arg712His | Cardiomyopathy, dilated 1Sl | Y | 2021 |
| *CACNA1D* | c.5876C>T, p.Ser1959Leu | Primary aldosteronism, seizures, and neurologic abnormalities; Sinoatrial node dysfunction and deafness | N |  |
| *GATA6* | c.1756C>T, p.Pro586Ser | Heart defects, congenital and other congenital anomalies, Tetralogy of Fallot, Conotruncal heart malformations, ASD 5, ASD9 | Y | 2019 |
| *NEK8* | c.1418-1G>A | Renal-Hepatic-Pancreatic dysplasia 2, Nephronophthisis 9 | Y | 2021 |
| *MRPS22* | c.732+1G>A; c.1009C>T, p.Gln337Ter | Combined oxidative phosphorylation deficiency 5 | Y | 2019 |
| *KMT2D* | c.8994dupT, p.Ala2999CysfsTer | Kabuki Syndrome 1 | Y | 2019 |
| *CHD7* | c.7252C>T, p.Arg2418Ter | CHARGE syndrome | Y | 2019 |
| *TSC2* | c.2410T>C, p.Cys804Arg | Tuberous Sclerosis | Y | 2019 |
| *SOX9* | c.1005>A, p.Trp335Ter | Campomelic dysplasia | Y | 2019 |
| *SMARCE1* | c.665C>G, p.Ala222Gly | Coffin-Siris syndrome 5 | Y | 2021 |
| *EHMT1* | c.3150G>A, p.Met1050lle | Kleefstra Syndrome 1 | Y | 2019 |
| *EFTUD2* | c.1705C>T, p.Arg569Ter | Mandibulofacial dysostosis,  Guion-Almeida type | Y | 2019 |
| *ADNP* | c.215dupA, p.Tyr719Ter | Helsmoortel-Van der AA syndrome | Y | 2019 |
| *EVC2* | c.2335G>T, p.Glu779Ter; c.2965_2967delCTC, p.L989del | Ellis-van Creveld syndrome | Y | 2019 |
| *PTPN11* | c.1510A>G, p.Met504Val | Noonan syndrome 1 | Y | 2019 |
| *CHD7/NOTCH1* | c.2176_2177delGA, p.Asp726LeufsTer12; c.1915G>C, p.Glu639Gln | CHARGE syndrome | Y | 2019 |
| *SMARCA4* | c.4795G>A, p.Glu1599Lys | Coffin-Siris syndrome 4 | Y | 2019 |
| *CACNA1D* | c.3271G>A, p.Val1091Met | Primary aldosteronism, seizures and neurologic abnormalities, sinoatrial node dysfunction and deafness | N |  |
| *SMARCA4* | c.3460C>T, p.Leu1154Phe | Coffin-Siris syndrome 4 | Y | 2019 |
| *FGFR2* | c.833G>T, p.Cys278Phe | Apert syndrome; Crouzon syndrome; Saethre-Chotzen syndrome; Pfeiffer syndrome; Jackson-Weiss Syndrome; Beare-Stevenson Cutis Gyrata syndrome; Lacrimoauriculodentodigital syndrome; Antley-Bixler syndrome; scaphocephaly maxillary retrusion and mental retardation; Bent bone dysplasia syndrome; gastric cancer | Y | 2019 |
| *MYH6* | c.3530G>T, p.Arg1177Leu | Familial hypertrophic cardiomyopathy 14 | Y | 2019 |
| *TSC2* | c.5238_5255del18, p.His1746_Arg1751del | Tuberous sclerosis 2 | Y | 2019 |
| *RAF1* | c.770C>T, p.Ser257Leu | Noonan syndrome 5 | Y | 2019 |
| *TSC1* | c.880G>A, p.Gly294Arg | Tuberous sclerosis 2 | Y | 2019 |
| *ISPD* | c.674delC (p.A225Dfs*21) c.1106 T >G (p.V369 G) | Walker-Warburg syndrome | Y | 2019 |
| *FGFR3* | c.1144 G > A (p.G382R) | Achondroplasia syndrome | Y | 2019 |
| *ROR2* | c.794 G>A (p.R265 H); c.1675 G > A (p.G559S) | Robinow syndrome | Y | 2019 |
| *COL2A1* | c.1151 G>T (p.G384 V) | Achondroplasia syndrome | Y | 2019 |
| *RPL11* | c.499C>T (p.Q167*) | Diamond-Blackfan anaemia | Y | 2019 |
| *COL1A1* | c.740delC (p.P247Lfs*18) | Osteogenesis Imperfecta | Y | 2019 |
| *TSC1* | c.2128C>T (p.Q710*) | Tuberous sclerosis | Y | 2019 |
| *COL1A1* | c.3230_3231delCT (p.P1077Rfs*18) | Osteogenesis Imperfecta | Y | 2019 |
| *L1CAM* | c.1322delG (p.G441Afs*72) | Hydrocephalus due to Aqueductal stenosis | Y | 2019 |
| *GHR* | c.497 G>A (p.G166E) | Laron dwarfism/growth hormone insensitivity | Y | 2024 |
| *DNAH5* | c.13774C>T (p.R4592*) | Ciliary dyskinesia with or without situs inversus | Y | 2019 |
| *KLHL40* | c.1516A>C (p.T506 P) | Nemaline myopathy 8 | Y | 2019 |
| *COL1A1* | c.1669-2A>G | Osteogenesis Imperfecta | Y | 2019 |
| *L1CAM* | c.551 G>A (p.R184Q) | Hydrocephalus due to Aqueductal stenosis | Y | 2019 |
| *COL1A1* | c.2155 G>A (p.G719S) | Osteogenesis Imperfecta | Y | 2019 |
| *NOTCH1* | c.5167 + 1G>A | Aortic valve disease 1 | Y | 2019 |
| *NPHP3* | c.424C>T (p.R142*) | Cystic dysplasia of the kidneys/nephronophthisis/pancreatic dysplasia | Y | 2019 |
| *COL2A1* | c.4082A>G (p.Y1361C) | Achondroplasia syndrome | Y | 2019 |
| *FGFR3* | c.1144 G>A (p.G382R) | Achondroplasia syndrome | Y | 2019 |
| *TSC2* | c.2764_2765delTT (p.L922Vfs*3) | Tuberous sclerosis-2 | Y | 2019 |
| *PKD1* | c.6571C>T; p.Arg2191Cys | ADPKD | Y | 2019 |
| *ACTA2* | c.536G>A: pR179H | Multisystemic smooth muscle dysfunction syndrome | Y | 2019 |
| *PKHD1* | c.8301del p.N2768fs*18; c.4481delp:N14947fs*6 | PKD, with or without hepatic disease | Y | 2019 |
| *PPM1D* | c.1434delC p.R2191C | Neurodevelopmental disorders syndrome | N |  |
| *SETD2* | c.4376C>T (p.R1459X) | Luscan-Lumish syndrome | Y | 2022 |
| *TMEM231* | c.525+1G>A; c.661C>T (p.R221X) | Meckel Syndrome | Y | 2019 |
| *PTPN1* | c.124A>G, p.T42A | Noonan Syndrome | Y | 2019 |
| *RAF1* | c.770C>T (p.S257L) | Noonan Syndrome | Y | 2019 |
| *PKHD1* | c.7994T>C, p.Lei2665Pro; c.5428G>T, p.Glu1810* |  | Y | 2019 |
| *COL1A1* | c.4280_4283delTTGA, p.Ile1427Asnfs*98 | Osteogenesis imperfecta; Caffey disease | Y | 2019 |
| *SBDS* | c.183_184delinsCT, p.Lys62*; c.258+2T>C | Shwachman-Diamond syndrome | Y | 2019 |
| *MUSK* | c.790C>T p.Arg264*, c.1003_1006delGTTT, p.Val335Phefs*24 | Fetal akinesia deformation sequence 1 | Y | 2019 |
| *HNF1B* | c.494G>A, p.Arg165His | Renal cysts and diabetes syndrome | Y | 2019 |
| *TSC2* | c.4258_4261TCOG, p.Ser1420Glyfs*55 | Tuberous sclerosis-2 | Y | 2019 |
| *TSC2* | c.4762C>T, p.Gln1588* | Tuberous sclerosis-2 | Y | 2019 |
| *TSC2* | c.4762C>T, p.Gln1588* | Tuberous sclerosis-2 | Y | 2019 |
| *RYR1* | c.6082C>T, p.Arg2028*; c.165+5G>A | Minicore myopathy with external ophthalmoplegia | Y | 2019 |
| *CHD7* | c.2881delG, p.Glu961Serfs*16 | CHARGE Syndrome | Y | 2019 |
| *ZIC2* | c.916 G>T, p.Glu306* | Holoprosencephaly 5 | Y | 2019 |
| *IARS1* | c.2420C>G, p.Pro807 Arg; c.2975A>G, p.Asn992Ser | Growth retardation, impaired intellectual development, hypotonia and hepatopathy | Y | 2019 |
| *CHD7* | c.7153C>T, p.Gln2385* | CHARGE syndrome | Y | 2019 |
| *PIGN* | c.963G>A and c.1859+1G>A | Multiple congenital anomalies; hypotonia; seizures syndrome 1 | Y | 2021 |
| *SOS1* | c.1297G>A (p.E433K) | Noonan Syndrome 4 | Y | 2019 |
| *ECE1* | c.1930G>A (p.G644R) | Cardiac defects, autonomic dysfunction | N |  |
| *DSTYK* | c.2125C>T | Congenital anomalies of kidney and urinary tract | Y | 2019 |
| *CHD7* | c.5428C>T; p.R1810X | CHARGE | Y | 2019 |
| *PEX1* | c.3205C>T (p.Q1069X), c.2097_2098insT (p.1700fs) | Heimler syndrome 1/Perioxisome biogenesis disorder 1A/1B | Y | 2019 |
| *TUBB3* | c.862G>A, (p.E288K) | Cortical dysplasia complex with other brain malformations, fibrosis of extraocular muscles | Y | 2021 |
| *TUBA1A* | c.55G>A, (p.A19T) | Lissencephaly 3 | Y | 2019 |
| *TUBB* | c.920C>T, p.P307L | Cortical dysplasia complex with other brain malformations, symmetric circumferential skin creases | Y | 2019 |
| *ACTB* | c.617G>A, (p.R206Q) | Baraitser-Winter syndrome | Y | 2019 |
| *HIVEP2/DNAH7* | c.2968_2971del (p.K990fs); c.8624G>A (p.T2875Q); c.4787_4788insA (p.Y1596X) | Intellectual developmental disorder, ciliary dyskinesia | Y/N | 2019 |
| *PDHA1* | c.1134dupG, (p.V378fs) | Pyruvate dehydrogenase E1 alpha deficiency | Y | 2019 |
| *TUBB3* | c.533C>T, (p.T178M) | Cortical dysplasia complex with other brain malformations, fibrosis of extraocular muscles | Y | 2021 |
| *CEP290* | c.6401T>C, (p.lle2134Thr), c.4195-1G>A | Joubert syndrome 5, Leber congenital amaurosis, Meckel syndrome, Senior-Loken syndrome | Y | 2019 |
| *TCTN1* | c.342-2A>Cc.898C>T: (p.R300X) | Joubert syndrome 13 | Y | 2019 |
| *TMEM67* | c.233G>A (p.C78Y); c.1717delA (p.N573fs) | Bardel-Biedl syndrome 14, COACH syndrome, Joubert syndrome 6, Meckel syndrome 3, Nephronophthisis 11 | Y | 2019 |
| *BBS10* | c.1241T>C (p.L414S), c.909_912del (p.S303fs) | Bardet-Biedl syndrome 10 | Y | 2019 |
| *EP300* | c.102_105del (p.G34fs) | Colorectal cancer, Menke-Hennekam syndrome 2, Rubinstein-Taybi syndrome 2 | Y | 2019 |
| *FAM20C* | c.956G>A (p.R319K); c.1528C>T (p.R510C) | Raine syndrome | Y | 2019 |
| *FLNB* | c.473G>C (p.A1578P) | Atelosteogenesis Type 1/III, Bomomerang dysplasia, Larsen syndrome, spondylocarpotarsal synostosis syndrome | Y | 2019 |
| *FRAS1* | c.370C>T (p.R124X) | Fraser syndrome 1 | Y | 2019 |
| *MNX1* | c.2T>C (p.M1T) | Currarino syndrome | Y | 2019 |
| *TGFBR1* | c.605_606insGAGAACTATTGT (p.A202delinsARTIV) | Loeys-Dietz syndrome | Y | 2019 |
| *RYR1* | c.784G>T (p.E262X) | Malignant hyperthermia susceptibility, congenital myopathy, Kingdenborough syndrome | Y | 2019 |
| *RYR1* | c.784G>T (p.E262X) |  | Y | 2019 |
| *RYR1* | c.328C>T (p.H110Y) c.14927T>C (p.L4976P) |  | Y | 2019 |

**Table S4** Pathogenic and likely pathogenic variants identified in virtual historical cohort and year they were added to NHS England R21 ‘green’ gene panel

| Gene | Variant | Syndrome | On current R21 gene list v5 (green) Y/N | Date added R21 panel green | Comments |
| --- | --- | --- | --- | --- | --- |
| *ATP7B* | c.2972C>T | Arthrogyrpoposis/Wilson disease | N |  | No clear evidence of association with FSA; Drury *et al* - considered to be IF |
| *MED13L* | c.4412_c.4413delTG, p.V1471Gfs* 7 | Neurodevelopmental delay and distinctive facial features with or without cardiac defects | Y | 2022 |  |
| *PLD1* | c.2447G>C | Cardiac Valvular Defect, developmental | Y | 2021 |  |
| *ABL1* | c.431A>G, p.His114Arg | Congenital heart defects and skeletal malformations syndrome | Y | 2021 |  |
| *MYH7* | c.2135G>A, p.Arg712His | Cardiomyopathy, dilated 1Sl | Y | 2021 |  |
| *CACNA1D* | c.5876C>T, p.Ser1959Leu | Primary aldosteronism, seizures, and neurologic abnormalities; Sinoatrial node dysfunction and deafness | N |  | Limited evidence association FSA; two cases Li et al, 2020 (amber FA panel); May need review |
| *NEK8* | c.1418-1G>A | Renal-Hepatic-Pancreatic dysplasia 2, Nephronophthisis 9 | Y | 2021 |  |
| *SMARCE1* | c.665C>G, p.Ala222Gly | Coffin-Siris syndrome 5 | Y | 2021 |  |
| *CACNA1D* | c.3271G>A, p.Val1091Met | Primary aldosteronism, seizures and neurologic abnormalities, sinoatrial node dysfunction and deafness | N |  | Limited evidence association FSA; two cases Li *et al*, 2020 (amber FA panel); May need review |
| *GHR* | c.497 G > A (p.G166E) | Laron dwarfism/growth hormon insensitivity | Y | 2024 |  |
| *PPM1D* | c.1434delC p.R2191C | Neurodevelopmental disorders syndrome | N |  | No clear evidence of association with FSA; Zhou *et al* - considered to be IF |
| *SETD2* | c.4376C>T (p.R1459X) | Luscan-Lumish syndrome | Y | 2022 |  |
| *PIGN* | c.963G>A and c.1859+1G>A | multiple congenital anomalies; hypotonia; seizures syndrome 1 | Y | 2021 |  |
| *ECE1* | c.1930G>A (p.G644R) | Cardiac defects, autonomic dysfunction | N |  | No clear gene-disease association demonstrated with relevance to FSA |
| *TUBB3* | c.862G>A, (p.E288K) | Cortical dysplasia complex with other brain malformations, fibrosis of extraocular muscles | Y | 2021 |  |
| *DNAH7* | c.2968_2971del (p.K990fs); c.8624G>A (p.T2875Q); c.4787_4788insA (p.Y1596X) | Intellectual developmental disorder, ciliary dyskinesia | N |  | No clear gene-disease association demonstrated with relevance to FSA |
| *TUBB3* | c.533C>T, (p.T178M) | Cortical dysplasia complex with other brain malformations, fibrosis of extraocular muscles | Y | 2021 |  |

**Table S5** Genes added sequentially to and genes missing from NHS England R21 ‘green’ gene panel

[FA, fetal anomaly; FSA, fetal structural anomaly; IF, incidental finding]

| Region/country with phenotypic criteria | NHS England | Spain | Canada (Ontario) | Canada (British Columbia) | Greece |
| --- | --- | --- | --- | --- | --- |
| NHS England | – | 0.512 | 0.663 | 0.366 | 0.723 |
| Spain, | 0.512 | – | 0.947 | 0.794 | 0.345 |
| Canada (Ontario) | 0.663 | 0.947 | – | 0.789 | 0.490 |
| Canada (British Columbia) | 0.366 | 0.794 | 0.789 | – | 0.244 |
| Greece | 0.723 | 0.345 | 0.490 | 0.244 | – |

**Table S6** Matrix of *P*-values for pairwise comparisons between summary area under receiver-operating-characteristics curve of international phenotypic eligibility criteria for prenatal exome sequencing

[P-values for pairwise comparisons between the areas under the summary receiver operating characteristics curves of each set of phenotypic criteria, estimated with 5,000 bootstrap samples.]

| Region/country with phenotypic criteria | NHS England | Spain | Canada (Ontario) | Canada (British Columbia) | Greece |
| --- | --- | --- | --- | --- | --- |
| NHS England | – | 0.093 | 0.551 | 0.175 | 0.066 |
| Spain | 0.093 | – | 0.031 | 0.927 | 0.754 |
| Canada (Ontario) | 0.551 | 0.031 | – | 0.070 | 0.023 |
| Canada (British Columbia) | 0.175 | 0.927 | 0.070 | – | 0.720 |
| Greece | 0.066 | 0.754 | 0.023 | 0.720 | – |

**Table S7** Matrix of *P*-values for pairwise comparisons between pooled sensitivities of international phenotypic eligibility criteria for prenatal exome sequencing

[P-values for pairwise comparisons between the pooled sensitivities of each set of phenotypic criteria, estimated using z-tests.]

| Region/country with phenotypic criteria | NHS England | Spain | Canada (Ontario) | Canada (British Columbia) | Greece |
| --- | --- | --- | --- | --- | --- |
| NHS England | – | 0.347 | 0.473 | 0.436 | 0.006 |
| Spain | 0.347 | – | 0.158 | 0.808 | 0.326 |
| Canada (Ontario) | 0.473 | 0.158 | – | 0.178 | < 0.001 |
| Canada (British Columbia) | 0.436 | 0.808 | 0.178 | – | 0.150 |
| Greece | 0.006 | 0.326 | < 0.001 | 0.150 | – |

**Table S8** Matrix of *P*-values for pairwise comparisons between pooled specificities of international phenotypic eligibility criteria for prenatal exome sequencing

[P-values for pairwise comparisons between the pooled specificities of each set of phenotypic criteria, estimated using z-tests.]

**References**

1. Aarabi M, Sniezek O, Jiang H, et al. Importance of complete phenotyping in prenatal whole exome sequencing. Hum Genet. 2018;137(2):175‐181
2. Boissel S, Fallet‐Bianco C, Chitayat D, et al. Genomic study of severe fetal anomalies and discovery of GREB1L mutations in renal agenesis. Genet Med. 2018;20(7):745‐753
3. Carss KJ, Hillman SC, Parthiban V, et al. Exome sequencing improves genetic diagnosis of structural fetal abnormalities revealed by ultrasound. Hum Mol Genet. 2014;23(12):3269‐3277.
4. Chen M, Chen J, Wang C, et al. Clinical application of medical exome sequencing for prenatal diagnosis of fetal structural anomalies. Eur JObstet Gynecol Reprod Biol. 2020;251:119‐124
5. Choy KW, Wang H, Shi M, et al. Prenatal diagnosis of fetuses with increased nuchal translucency by genome sequencing analysis. Front Genet. 2019;10.
6. Drury S, Williams H, Trump N, et al. Exome sequencing for prenatal diagnosis of fetuses with sonographic abnormalities. Prenat Diagn.2015;35(10):1010‐1017
7. Fu F, Li R, Li Y, et al. Whole exome sequencing as a diagnostic adjunct to clinical testing in fetuses with structural abnormalities. Ultrasound Obstet Gynecol. 2018;51(4):493‐502
8. Heide S, Spentchian M, Valence S, et al. Prenatal exome sequencing in 65 fetuses with abnormality of the corpus callosum: contribution to further diagnostic delineation. Genet Med. 2020;22(11):1887‐1891.
9. Lefebvre M, Bruel A‐L, Tisserant E, et al. Genotype‐first in a cohort of 95 fetuses with multiple congenital abnormalities: when exome sequencing reveals unexpected fetal phenotype‐genotype correlations. J Med Genet. 2021;58(6):400‐413.
10. Lei T‐Y, Fu F, Li R, et al. Whole‐exome sequencing in the evaluation of fetal congenital anomalies of the kidney and urinary tract detected by ultrasonography. Prenat Diagn. 2020
11. Lei L, Zhou L, Xiong JJ. Whole‐exome sequencing increases the diagnostic rate for prenatal fetal structural anomalies. Eur J Med Genet. 2021;64(9):104288.
12. Leung GKC, Mak CCY, Fung JLF, et al. Identifying the genetic causes for prenatally diagnosed structural congenital anomalies (SCAs) by whole‐exome sequencing (WES). BMC Med Genomics. 2018;11(1):93.
13. Li R, Fu F, Yu Q, et al. Prenatal exome sequencing in fetuses with congenital heart defects. Clin Genet. 2020.
14. Lord J, McMullan DJ, Eberhardt RY, et al. Prenatal exome sequencing analysis in fetal structural anomalies detected by ultrasonography (PAGE): a cohort study. Lancet. 2019;393(10173):747‐757.
15. Petrovski S, Aggarwal V, Giordano JL, et al. Whole‐exome sequencing in the evaluation of fetal structural anomalies: a prospective cohort study. Lancet. 2019;393(10173):758‐767.
16. Qi Q, Jiang Y, Zhou X, et al. Simultaneous detection of CNVs and SNVs improves the diagnostic yield of fetuses with ultrasound anomalies and normal karyotypes. Genes (Basel). 2020;11(12):1397.
17. Qiao F, Wang Y, Zhang C, et al. Comprehensive evaluation of genetic variants using chromosomal microarray analysis and exome sequencing in fetuses with congenital heart defect. Ultrasound Obstet Gynecol. 2021;58(3):377‐387
18. Quinlan‐Jones E, Lord J, Williams D, et al. Molecular autopsy by trio exome sequencing (ES) and postmortem examination in fetuses and neonates with prenatally identified structural anomalies. Genet Med. 2019;21(5):1065‐1073.
19. Sun H, Yi T, Hao X, et al. Contribution of single‐gene defects to congenital cardiac left‐sided lesions in the prenatal setting. Ultra-sound Obstet Gynecol. 2020;56(2):225‐232
20. Xue S, Yan H, Chen J, et al. Genetic examination for fetuses with increased fetal nuchal translucency by genomic technology. Cytogenet Genome Res. 2020;160(2):57‐62
21. Yang X, Huang L, Pan M, et al. Exome sequencing improves genetic diagnosis of fetal increased nuchal translucency. Prenat Diagn.2020;5789
22. Zhou J, Yang Z, Sun J, et al. Whole genome sequencing in the evaluation of fetal structural anomalies: a parallel test with chromosomal microarray plus whole exome sequencing. Genes (Basel).2021;12(3):1‐14
23. Zhou X, Wang Y, Shao B, et al. Molecular diagnostic in fetuses with isolated congenital anomalies of the kidney and urinary tract by whole‐exome sequencing. J Clin Lab Anal. 2020;e23480
24. Mellis R, Oprych K, Scotchman E, Hill M, Chitty LS. Diagnostic yield of exome sequencing for prenatal diagnosis of fetal structural anomalies: A systematic review and meta-analysis. Prenat Diagn. 2022 May;42(6):662-685. doi: 10.1002/pd.6115. Epub 2022 May 7. PMID: 35170059; PMCID: PMC9325531.
