# Supplementary material for: Performance of international phenotypic criteria for prenatal exome sequencing: systematic review and comparative diagnostic accuracy study using historical individual participant data
Source: Ultrasound Obstet Gynecol. 2025 Jul 8;66(3):282–9. doi: 10.1002/uog.29290 (PMC12401499; doi:10.1002/uog.29290)
Supplement: Supplementary file 3 — Appendix S1 Fetal phenotypic eligibility criteria for prenatal exome sequencing in Ontario, Canada [file UOG-66-282-s003.pdf]

Ontario

Ministry of Health

OHIP,
Pharmaceuticals and Devices Division
Laboratories and Diagnostics Branch

438 University Ave, 4th Floor
Toronto ON, M7A 1N3
Telephone: 416-326-4444

Ministère de la Santé

Division de l'Assurance-santé, des produits pharmaceutiques et des appareils et accessoires fonctionnels
Direction des laboratoires et des services diagnostiques

438, av. University, 4e étage
Toronto ON, M7A 1N3
Téléphone: 416-326-4444

ELIGIBILITY CHECKLIST FOR PRIOR APPROVAL OF OUT-OF-COUNTRY/OUT-OF-PROVINCE CLINICAL PRENATAL WHOLE EXOME SEQUENCING

The Ministry of Health (ministry) requires that applications to the Out-of-Country/Out-of-Province Prior Approval (OOC/OOP PA) program for clinical prenatal whole exome sequencing (WES) be accompanied by a completed copy of the enclosed eligibility checklist and any relevant clinical history information. Incomplete applications will delay the review and adjudication of your funding request.

In alignment with the clinical prenatal guidance document developed by Canadian experts in the field of prenatal and molecular genetics, this checklist outlines specific clinical criteria for prenatal WES testing to help assess whether the applicable regulatory requirements under the *Health Insurance Act* are met. Expert opinion suggests that WES may be beneficial in the prenatal assessment of certain structural fetal anomalies, as long as the timeline for obtaining results is sufficient for making changes in pregnancy and/or neonatal management.

The gestational age, timeline required for results, turnaround time and cost should be taken into account when requesting the type of testing approach (urgent or semi-urgent and non-urgent). Consideration should be given to ordering this testing in the most economical way, while ensuring the length of time for obtaining results is sufficient to inform the patient's management options. Note that these are interim guidelines that will evolve as more data is generated on the clinical utility of this testing.

Recommended WES Approach

- Trios are the recommended prenatal WES strategy if both biological parents are available for testing.
- Duos are recommended in cases where there is only one first-degree relative available for testing (i.e., a parent or an affected sibling if a parent is not available).
- Proband-only cases are not recommended for prenatal WES at this time. A comprehensive panel is the preferred option as the diagnostic rate for WES done during the pregnancy on proband-only cases is anticipated to be lower. As more data emerges, these recommendations will be reassessed.

Deciding Between WES and a Targeted Gene Panel

If the fetus has a phenotype highly specific to a known genetic condition for which an optimized genetic panel exists or for which all known gene-disease associations could be assessed, the targeted gene panel should be given priority.

If a comprehensive gene panel has been completed and did not identify a causative pathogenic gene variant but there is a strong argument to be made for prenatal WES, please include this rationale in your application form for prenatal WES.

Chromosomal Microarray Requirement:

An application for clinical prenatal WES should only be completed for an affected pregnancy after chromosomal microarray (CMA) has been performed and where the array results did not identify any pathogenic variants explaining the phenotype.

a. If a chromosomal microarray is in progress at the time of submission, please indicate this in section 4B of the enclosed checklist. A conditional approval will be granted if the patient qualifies pending the results of the microarray.
b. Upon conditional approval, the sample can be sent to the testing laboratory with instructions to hold the sample until a final decision letter is received from the ministry.
c. Results of the CMA, once received, should be communicated to the ministry's OOC/OOP PA Program by submitting the additional information through the e-form as shown below:
    i. Check the box indicating that you are providing additional information for a previous submission and fill in the PA number and physician email, then click "Next".

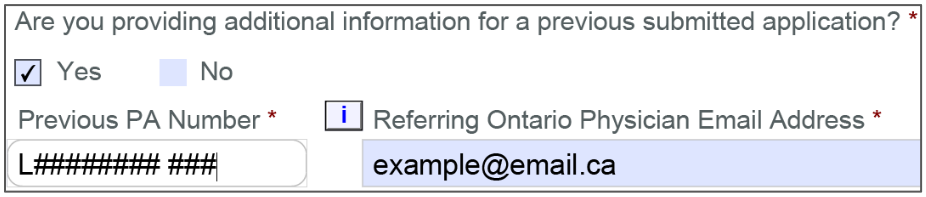
Are you providing additional information for a previous submitted application? *
☑ Yes ☐ No
Previous PA Number * | Referring Ontario Physician Email Address *
L######## ### | example@email.ca

    ii. Click "Add File" and attach this document with the template in Appendix 1 completed.
    iii. Complete the attestation section of the e-form, review and then submit.
    iv. A final decision letter will be issued once your submission is processed. If the CMA results are conclusive and, as a result, the final decision letter is a denial of prior approval, please follow up with the laboratory to withdraw your request.

Options if WES Results are Unrevealing:

If the clinical presentation of your patient is not explained after clinical prenatal WES, you can consider the following:

- Request re-analysis (if additional phenotypic data is identified postnatally or no less than 1 year after initial analysis if no additional phenotypic data is known)
- With appropriate consent, share your patient's phenotypic data, candidate gene and/or WES data with one of the many global sharing initiatives for unsolved rare diseases (e.g. www.phenomecentral.org)

The following checklist is for samples submitted on the current pregnancy only and cannot be used for prior approval applications for WES where the sample is from a previous pregnancy/stillbirth/living sibling requesting a rapid/express turn-around-time for an ongoing pregnancy. Other clinical situations requiring WES can be submitted to Genome-wide Sequencing Ontario (gsontario.ca) for in-province testing on postnatal and non-urgent samples, or to the ministry's OOC/OOP Prior Approval program with the consult notes and rationale, if Ontario testing is not suitable for the patient.

CLINICAL PRENATAL WHOLE EXOME SEQUENCING (WES) CHECKLIST

This document contains private and confidential information.

Please complete the eligibility checklist below and upload the completed version to Part 5 of your OOC/OOP PA application e-Form.

Patient Name: ____ (herein referred to as "the patient")

SECTION 1: Analysis and Approach

[See "Laboratory Analysis and Approach" section for guidance.]

I am requesting a prenatal WES on a fetal sample for:

- ☐ Urgent or semi-urgent prenatal/imminent postnatal management *(requiring a rapid turnaround time for pregnancy management decisions or immediate postnatal management)*
- ☐ Non-urgent postnatal management *(WES ordered during the pregnancy to inform postnatal management requiring a less urgent turnaround time)*

I am requesting a:

- ☐ Trio *(Recommended option, best for interpretation)*
- ☐ Duo *(Recommended for cases where only one first degree relative is unavailable for testing)*

THE CRITERIA LISTED IN EITHER SECTION 2 <u>OR</u> SECTION 3 MUST BE MET

SECTION 2: Single Organ System Anomalies Criteria

With the exception of hydrops fetalis (see definition below), single organ system anomalies will not be approved for funding for clinical prenatal WES at this time. Two anomalies in a single organ (i.e. brain) or a skeletal dysplasia with two different skeletal findings would also fall under this category. This criterion will be revisited, as new evidence emerges.

A. I confirm that:

Yes	No	
☐	☐	The fetus is presenting with hydrops fetalis, defined as accumulation of fluid in two or more fetal compartments including increased nuchal translucency (≥3.5 mm)/cystic hygroma, pleural effusion, pericardial effusion, ascites, skin edema.<br>*(Please note: Isolated increased nuchal translucency or isolated cystic hygroma are not eligible findings on their own).*

SECTION 3: Multiple Congenital Anomalies Criteria

A. I confirm that (must meet ≥ 2 items):

Yes	No	
☐	☐	The fetus is presenting with multiple congenital anomalies, at least 2 of which are major anomalies and involve at least 2 different systems (e.g., brain and skeletal).
☐	☐	Differential diagnosis includes ≥ 2 well defined conditions requiring evaluation by multiple targeted gene panels.
☐	☐	The fetus is presenting with one major anomaly and IUGR (by objective measure) /oligohydramnios/polyhydramnios *(when not explained by maternal/teratogenic factors/incorrect dating or IUGR arising from placental factors)*.

B. I confirm that, to the best of my knowledge, the fetal anomalies are not (must apply to all items):

Yes ☐ No ☐

- Multiple congenital anomalies with gastroschisis as one of the major anomalies.
- An isolated neural tube defect with their associated anomalies (e.g. ventriculomegaly, clubfeet).
- Soft markers (including mild ventriculomegaly) included as one of the major organ system anomalies.
- Anomalies explained by maternal/teratogenic/placental factors.
- Amniotic bands.
- A highly recognizable pattern specific to a known genetic condition for which an optimized genetic panel exists or for which all known gene-disease associations could be assessed. (If so, then the targeted gene panel should be given priority assuming it is more sensitive (e.g. Noonan spectrum disorders)).

SECTION 4: Additional Criteria

A. I confirm that (must meet all items):

Yes ☐ No ☐

- Detailed phenotypic characterization using high resolution ultrasound and/or fetal MRI was performed prior to testing.
- Pretest genetic counselling and informed consent has been completed and the patient has indicated that this additional information will help in pregnancy management/decision making **OR** The patient and the clinical team have indicated that this information may impact immediate postnatal management.
- The timeline of results will be sufficient for pregnancy management options or postnatal clinical management.

B. I confirm that (must meet 1 item):

Yes	No	
☐	☐	Chromosomal microarray has been completed and no causative variant is identified on microarray explaining the fetus's phenotype.
☐	☐	Chromosomal microarray is in progress and this OOC/OOP PA application will be withdrawn if a causative variant is identified on microarray explaining the fetus's phenotype. Results of the CMA, once received, will be communicated to the ministry's OOC/OOP PA with the request to either continue or cancel the test application (see Appendix 1).

C. I confirm that:

Yes	No	
☐	☐	• All cases where funding for prenatal WES has been applied for will be entered into the BORN Information System by the clinic applying for funding, including declined cases.

SECTION 5: Declarations and Signatures

By signing this checklist below, each Ontario physician named below confirms that:

- The information provided in this checklist is true, correct, and complete.
- It is an offence under subsection 43(3) of the *Health Insurance Act* to give information that the physician knew or ought to have known is false in an application to the General Manager.
- In the case of the physician specialist named below, the physician meets the following criteria:
  - the physician treats the medical condition(s) described in this checklist; and
  - the physician practices in medical genetics on an ongoing basis; and
  - the physician holds one of the appropriate certifications to qualify as a "specialist" as defined in Ontario's Schedule of Benefits for Physician Services (please refer to the "Definitions" section under the General Preamble)

Instructions:

- Where the Ontario physician submitting the OOC/OOP PA application meets the qualifications for an Ontario physician specializing in medical genetics set out in the declarations above, then the physician shall enter their name twice below.
- Where the Ontario physician submitting the OOC/OOP PA application does not meet qualifications for an Ontario physician specializing in medical genetics set out in the declarations above, then the physician shall enter their name beside “Physician Name” and the geneticist who was consulted on the application shall enter their name beside “Geneticist Name”.

Physician Name: ___________________________________ Date:_________________________

By typing your name into the signature field, you agree that you are signing this form electronically and that your electronic signature is the legal equivalent of your manual signature on this form.

Geneticist Name: _________________________________ Date:_________________________

By typing your name into the signature field, you agree that you are signing this form electronically and that your electronic signature is the legal equivalent of your manual signature on this form.

Please attach a completed copy of this eligibility checklist and any relevant clinical history information to Part 5 of the ministry’s OOC/OOP Prior Approval Application e-Form.

Clear Form

Appendix 1: Chromosomal Microarray Results Confirmation

If your application was granted conditional approval pending CMA results and you have now received the results, please complete the form below and submit it to the OOC/OOP PA program through the online application e-form (instructions on 2).

Patient Name: ______________________________________

Chromosomal Microarray Result Update:

A. I confirm that (select 1 option):

☐ Chromosomal microarray has been completed and no causative variant is identified on the microarray explaining the fetus's phenotype.
Please, continue with the test application.

☐ Chromosomal microarray has been completed and a causative variant is identified on microarray explaining the fetus's phenotype.
Please, cancel the test application.

Physician Name: ______________________________ Date:______________________

Clear Form
